# Transcript Profiling of MIKCc MADS-Box Genes Reveals Conserved and Novel Roles in Barley Inflorescence Development

**DOI:** 10.3389/fpls.2021.705286

**Published:** 2021-09-01

**Authors:** Hendrik N. J. Kuijer, Neil J. Shirley, Shi F. Khor, Jin Shi, Julian Schwerdt, Dabing Zhang, Gang Li, Rachel A. Burton

**Affiliations:** ^1^School of Agriculture Food and Wine, University of Adelaide, Glen Osmond, SA, Australia; ^2^Joint International Research Laboratory of Metabolic and Developmental Sciences, Shanghai Jiao Tong University-University of Adelaide Joint Centre for Agriculture and Health, School of Life Sciences and Biotechnology, Shanghai Jiao Tong University, Shanghai, China; ^3^School of Life Sciences and Engineering, Southwest University of Science and Technology, Mianyang, China

**Keywords:** barley, inflorescence, floral organs, meristems, MADS, transcript profiling, ABC model, floret

## Abstract

MADS-box genes have a wide range of functions in plant reproductive development and grain production. The ABCDE model of floral organ development shows that MADS-box genes are central players in these events in dicotyledonous plants but the applicability of this model remains largely unknown in many grass crops. Here, we show that transcript analysis of all MIKCc MADS-box genes through barley (*Hordeum vulgare* L.) inflorescence development reveals co-expression groups that can be linked to developmental events. Thirty-four MIKCc MADS-box genes were identified in the barley genome and single-nucleotide polymorphism (SNP) scanning of 22,626 barley varieties revealed that the natural variation in the coding regions of these genes is low and the sequences have been extremely conserved during barley domestication. More detailed transcript analysis showed that MADS-box genes are generally expressed at key inflorescence developmental phases and across various floral organs in barley, as predicted by the ABCDE model. However, expression patterns of some *MADS* genes, for example *HvMADS58* (AGAMOUS subfamily) and *HvMADS34* (SEPALLATA subfamily), clearly deviate from predicted patterns. This places them outside the scope of the classical ABCDE model of floral development and demonstrates that the central tenet of antagonism between A- and C-class gene expression in the ABC model of other plants does not occur in barley. Co-expression across three correlation sets showed that specifically grouped members of the barley MIKCc MADS-box genes are likely to be involved in developmental events driving inflorescence meristem initiation, floral meristem identity and floral organ determination. Based on these observations, we propose a potential floral ABCDE working model in barley, where the classic model is generally upheld, but that also provides new insights into the role of MIKCc MADS-box genes in the developing barley inflorescence.

## Introduction

Flowers are often composed of four different floral organs organised in concentric whorls numbered from peripheral to central position. The outer whorls are sepals and petals in many dicots, including the model plant *Arabidopsis*, and lemma/palea and lodicules in grasses, while the inner whorls contain the male reproductive organs, the stamens, in the third whorl and the female organs, the carpels, in the fourth whorl (Ciaffi et al., 2011). Genetic studies have identified a large number of regulatory genes that control the specification of these distinct floral organs in plants (Alvarez-Buylla et al., 2010). The ABCDE model, originally proposed for *Arabidopsis* and *Antirrhinum majus*, associates the developmental determination of specific flower organs with the combinatorial activity of several classes of homeotic selector genes, most of which encode MIKCc MADS domain developmental transcription factors. Those MIKCc genes that function in the ABCDE model are divided into A-, B-, C-, D- and E-classes, which correspond with the AP1, AP3/PI, AG(C), AG(D) and SEP clades, respectively. A- and E-class genes determine the first whorl organs; A-, B- and E-class genes determine the second whorl; B-, C- and E-class genes control the third whorl; and C- and E-class genes specify the fourth whorl. D- and E-class genes are involved in ovule development within the carpel. Individual genes within a class usually act redundantly with each other in some roles, so that mutation of single members often leads to a subtle, absent or incomplete phenotype (Coen and Meyerowitz, 1991; Angenent et al., 1995; Pelaz et al., 2000). Studies demonstrate the conservation of gene homologues underlying the ABCDE model across most flowering plants, with only the AG subfamily (C-class) being present in gymnosperms (Theissen et al., 2016; Chen et al., 2017), suggesting the regulatory principles of some of these clades have been conserved during flower evolution.

The MADS-box genes have been divided into two subgroups: Type I and Type II, which are present in plants, animals and fungi (Kwantes et al., 2012; Chen et al., 2017). The encoded proteins cooperatively bind to DNA at conserved CArG boxes [CC(A/T)6GG] or [C(A/T)8G] to regulate gene expression (Theissen and Saedler, 2001). In plants, Type II MADS-box genes are called MIKC type, including MIKCc and MIKC* sub-types, an acronym of the four different domains that have been identified in all genes of this type. These transcription factors contain the conserved MADS DNA-binding domain (M), Intervening domain (I), Keratin-like domain (K) and the C-terminal domain (C), while the small c stands for classic. The closest relatives are the MIKC* genes, with the α-, β- and γ-MADS box genes completing the MADS family (Kwantes et al., 2012; Smaczniak et al., 2012). Within the MIKCc family, there are more clades than just those associated with the ABCDE model, such as the SVP-like floral repressors and the SOC1-like floral promotors (Becker and Theißen, 2003). The Type I lineage contains genes with simpler gene structure and lacking a Keratin-like domain. Their function is generally not well understood yet in plants, with some exceptions (Colombo et al., 2008; Callens et al., 2018). Additionally, MADS-box genes have also been reported to play an important role in abiotic stress, thermal regulation and plastic developmental responses in plants (Castelán-Muñoz et al., 2019; Li et al., 2021).

While the MIKCc clades and their roles in inflorescence development are generally conserved, the individual genes within a class often show no direct homology between grasses and *Arabidopsis*, making them co-orthologues (Ciaffi et al., 2011). More difficult to identify in grasses is the FLC-clade, which governs vernalisation and flowering time in *Arabidopsis* (Becker and Theißen, 2003). The FLC-clade genes in grasses are truncated and therefore could only be correctly classified using synteny and phylogenetic reconstruction (Ruelens et al., 2013). Present in grasses, but not in eudicots, is the OsMADS32 class, which is loosely related to B-class genes (Ciaffi et al., 2011).

The putative 11 MIKC-type MADS-box genes from the last common ancestor of monocots and eudicots increased to at least 24 genes in the last common ancestor of rice, wheat and maize (Bremer, 2002; Ciaffi et al., 2011). During this time of duplication and diversification in the MIKC family, the complex grass inflorescence and floral structures of the Poaceae family evolved. Changes in the copy number and expression pattern of MADS-box genes are closely associated with the morphological diversification of grass inflorescences (Ciaffi et al., 2011).

Generally, the ABCDE model of floral organogenesis can be applied to grasses as well. Functional studies in rice highlight mostly the homeotic changes defined by the model for predicted A-class genes (Wu et al., 2017), B-class genes (Nagasawa et al., 2003; Yadav et al., 2007; Yao et al., 2008), C- and D-class genes (Yamaguchi et al., 2006; Dreni et al., 2011) and E-class genes (Cui et al., 2010; Gao et al., 2010; Wu et al., 2018). The expression pattern and timing of MIKCc genes in other grasses indicate this likely extends to the whole grass family (Digel et al., 2015; Harrop et al., 2016; Feng et al., 2017; Callens et al., 2018; Zhu et al., 2018; Liu et al., 2020; Schilling et al., 2020).

A major determinant of floral organogenisis in grasses outside of the MADS-box genes is the YABBY family gene *DROOPING LEAF* (*DL*) which is involved in the regulation of carpel specification in rice (Nagasawa et al., 2003; Yamaguchi et al., 2004). Expression of *DL* orthologues in the carpel of maize and wheat is required to suppress the expression of B-class genes and is thus essential for floral organ specification according to the ABCDE model. Conserved expression suggests that DL function in carpel specification is a common feature in grasses (Bommert et al., 2005). While *CRABS CLAW*, the *Arabidopsis* co-orthologue of *DL*, has a function in carpel development, there is no homeotic change to the carpel identity in its absence (Bowman and Smyth, 1999), indicating a divergence in floral organogenesis between eudicots and grasses.

Some MIKCc genes associated with the ABCDE model have adopted additional roles in grasses, like the AP1 clade gene *HvMADS14* which is a vernalisation integrator in barley (Trevaskis et al., 2007a), expression of which is important for floral transition onset. Alternatively in rice, OsMADS34 has been shown to modulate inflorescence branching (Gao et al., 2010; Kobayashi et al., 2010), which is not canonically an E-class role.

The Triticeae crop barley has a simple branchless spike-type inflorescence. During early development, the inflorescence meristem differentiates several spikelet ridges, and each ridge develops a determinate triple spikelet meristem, which in turn gives three spikelet meristems (SM; Wang et al., 2021). Each SM produces one floral meristem, always resulting in three single-flowered spikelets per rachis node (Koppolu and Schnurbusch, 2019). Inflorescence development in barley, as well as wheat, can be divided into stages by examining the development of the inflorescence meristem and noting the emergence and shape of the floral, spikelet and floret meristems followed by the sequential initiation and growth of the floral organs (Waddington et al., 1983). These Waddington stages range from the transition of the vegetative to the reproductive meristem at W1, to pollination or anthesis at W10, and include a series of developmental and cellular events. However, the transcription and regulation of ABCDE model components in barley inflorescence development and floret formation still remain unknown. Here, we performed transcript analysis of MIKCc genes through inflorescence developmental stages and in individual floral organs by quantitative reverse transcription PCR (RT-qPCR). Our findings reveal that the ABCDE model can be mostly applied to barley, while deviations point to interesting adaptations that can reveal more about inflorescence development in grasses.

## Materials and Methods

### Identification of MIKCc Genes

Barley MIKCc MADS-box genes were identified by name and BLAST searches, using rice homologues, in the HORVU data set^1^ using Geneious software version 8.1.3 (Biomatters). Additional genes, and more accurate coding sequences, were found using an online tBLASTn search of transcript data at NCBI.^2^ Previously identified MADS-box genes annotated as MADS-box proteins in the Uniprot database,^3^ IPK Gatersleben and NCBI databases were categorised using the PlantTFDB as follow-up analysis for MIKC-type MADS-box members (Jin et al., 2014; Mascher et al., 2017; Monat et al., 2019). Where no known (complete) transcript sequences were available, the FGENESH+ protein-based gene prediction tool (Solovyev, 2007) was used to identify the most likely transcripts. Genes were named after their rice homologues, rather than the previous names used in barley, to standardise naming and make functional comparisons to other grasses easier (Table 1).

**Table 1:** The MIKCc MADS-box protein family in barley compared to rice.

	*Arabidopsis*	*Oryza* name	*Oryza* ID	Barley ID Morex1	Alt sequence	Barley ID Morex2	Legacy barley names
A-class	AP1, CAL, FUL	MADS14	Os03g0752800	HORVU5Hr1G095630.3		HORVU.MOREX.r2.5HG0424650.1	BM5, VRN1
				HORVU1Hr1G047550.1		HORVU.MOREX.r2.1HG0039100.1	‘HvAP1b’
		MADS15	OS07G0108900	HORVU2Hr1G063800.7	AK249833.1	HORVU.MOREX.r2.2HG0129220.1	BM8
		MADS18	OS07G0605200	^*^HORVU0Hr1G003020.3	AK361227.1	HORVU.MOREX.r2.2HG0105150.1	BM3
		MADS20	OS12G0501700	No equivalent			
B-class	PI	MADS2	OS01G0883100	HORVU3Hr1G091000.8		HORVU.MOREX.r2.3HG0256620.1	
		MADS4	OS05G0423400	HORVU1Hr1G063620.2		HORVU.MOREX.r2.1HG0051900.1	
	AP3	MADS16	OS06G0712700	^*^HORVU7Hr1G091210.4	AK373398.1	HORVU.MOREX.r2.7HG0598100.1	
C-class	AG, SHP1/2, STK	MADS3	OS01G0201700	HORVU3Hr1G026650.1		HORVU.MOREX.r2.3HG0202320.1	HvAG1
		MADS58	OS05G0203800	HORVU1Hr1G029220.1		HORVU.MOREX.r2.1HG0024570.1	HvAG2
D-class		MADS13	OS12G0207000	HORVU1Hr1G023620.1		HORVU.MOREX.r2.1HG0019750.1	
		MADS21	OS01G0886200	HORVU1Hr1G064150.2		HORVU.MOREX.r2.1HG0052300.1	
E-class	SEP1/2/4	MADS1	OS03G0215400	HORVU4Hr1G067680.2		HORVU.MOREX.r2.4HG0329790.1	HvMADS7
		MADS5	OS06G0162800	HORVU7Hr1G025700.6		HORVU.MOREX.r2.7HG0543420.1	
		MADS34	OS03G0753100	HORVU5Hr1G095710.1		HORVU.MOREX.r2.5HG0424690.1	
	SEP3	MADS7	OS08G0531700	HORVU7Hr1G054220.1		HORVU.MOREX.r2.7HG0567840.1	
		MADS8	OS09G0507200	HORVU5Hr1G076400.1		HORVU.MOREX.r2.5HG0409590.1	M9
AGL6	AGL6/13	MADS6	OS02G0682200	HORVU6Hr1G066140.9		HORVU.MOREX.r2.6HG0500990.1	AGL6
		MADS17	OS04G0580700	No equivalent			
	FCL1/2, AGL27/31	No equivalent in rice/barley					
	AGL14/19/42, SOC1	MADS50	OS03G0122600	No horvu number	AK368348.1	HORVU.MOREX.r2.4HG0343680.1	SOC1-1
		MADS56	OS10G0536100	^*^HORVU1Hr1G051660.8	JN673265	^*^HORVU.MOREX.r2.1HG0042540.1	SOC1-L
	AGL24, SVP(AGL22)	MADS22	OS02G0761000	HORVU6Hr1G077300.1		HORVU.MOREX.r2.6HG0511230.1	BM10
		MADS55	OS06G0217300	^*^HORVU7Hr1G036130.1	AK356695.1	HORVU.MOREX.r2.7HG0551090.1	VRT2
		MADS47	OS03G0186600	HORVU4Hr1G077850.3		HORVU.MOREX.r2.4HG0338120.1	BM1
	AGL12	MADS26	OS08G0112700	HORVU7Hr1G076310.14	AK370468.1	HORVU.MOREX.r2.7HG0585040.1	
		MADS33	OS12G0206800	No horvu number	AK250031.1	HORVU.MOREX.r2.2HG0140850.1	
	AGL16/17/44/21	MADS23	OS08G0431900	HORVU1Hr1G008290.1		HORVU.MOREX.r2.1HG0006360.1	
				HORVU1HR1G008300.3		HORVU.MOREX.r2.1HG0006350.1	M23b
		MADS25	OS04G0304400	M25a HORVU5Hr1G000480.1		HORVU.MOREX.r2.5HG0349390.1	
				M25b HORVU5Hr1G000370.3		^*^HORVU.MOREX.r2.5HG0349480.1	
				M25c HORVU7Hr1G023940.2		HORVU.MOREX.r2.7HG0541840.1	
				M25d HORVU7Hr1G024000.1		HORVU.MOREX.r2.7HG0541900.1	
		MADS27	OS02G0579600	HORVU2Hr1G080490.1		HORVU.MOREX.r2.2HG0143360.1	
		MADS57	OS02G0731200	^*^HORVU6Hr1G073040.13	AK363243.1	HORVU.MOREX.r2.6HG0507700.1	
B-sister	ABS	MADS29	OS02G0170300	HORVU6Hr1G032220.8		HORVU.MOREX.r2.6HG0473980.1	
		MADS30	OS06G0667200	HORVU7Hr1G108280.4	AK375718	HORVU.MOREX.r2.7HG0611760.1	
		MADS31	OS04G0614100	HORVU2Hr1G098930.2		^*^HORVU.MOREX.r2.2HG0158040.1	
	No At equivalent	MADS32	OS01G0726400	HORVU3Hr1G068900.3		^*^HORVU.MOREX.r2.3HG0237490.1	

The *Arabidopsis* co-orthologues are given per protein class where available, as the relation within classes is generally not homologous.

* An incomplete sequence or protein model.

### Phylogenetic Analysis

MIKCc MADS-box proteins from *Arabidopsis*, rice, sorghum and *Brachypodium* were collected from published data (Arora et al., 2007; Wei et al., 2014). The sequences obtained were aligned with previously identified barley MIKCc MADS-box proteins using the MUSCLE algorithm before manual inspection and minor adjustments (Edgar, 2004). The IQ-TREE web server was used to create a maximum likelihood phylogenetic tree (Trifinopoulos et al., 2016). JTT+I+G4 was selected as the best model and bootstrap was set at 1,000 replicates.

### SNP Analysis

A list of barley single-nucleotide polymorphisms (SNPs) was compiled using the comprehensive SNP database, recently made accessible at IPK Gatersleben^4^ and the barley pan-genome sets (Milner et al., 2019; Jayakodi et al., 2020).^5^ Gene locations in the SNP-browser were found by HORVU number where available, otherwise by position on the chromosome. Exon and amino acid changes were assessed by comparison to an alignment of cDNA sequences and chromosome fragments in Geneious 8.1.3 (Biomatters Ltd). Pan-genome predicted CDS sequences were extracted and assessed by multiple alignment in EUGENE (UniPro).^6^ Rice SNPs were collected using the online interface of the SNP database (Mansueto et al., 2017).^7^

### Inflorescence Tissue Sampling

*Hordeum vulgare* L. variety Golden Promise was grown in a controlled environment room with 16h light at 15°C day and 10°C night temperatures, at 70% humidity, in 8cm square pots containing coco-peat standing in closed trays and watered from below every 2days. A midday light maximum of 500μmole photons m^−2^ s^−1^ was used. Inflorescence tissue samples were taken from the main stem and examined under a dissecting microscope. Immature spikes exactly matching the desired Waddington stage (Waddington et al., 1983) were immediately frozen in liquid nitrogen and stored at −80°C.

For the W1 stage, where the meristem height was less than 1mm, 30 meristems were taken per sample. To capture transcript changes through pollination, one additional stage was introduced, called W10.5, which was taken 3days after pollination. One sample represents one biological replicate, for which 25 individual meristems (IM) were collected at W1.5, 20 IM at W2, 15 IM at W2.5, 12 IM at W3.5, 10 IM at W4, 8 IM at W4.5, 6 IM at W6.5 and 5 IM at W8.5. At W9.5 and W10.5, five separate single IM were taken, and combined at a later stage, such that each final sample comes from at least five individual plants.

Additionally, floral organ samples were taken from five different plants at Waddington stage 9.5. Twelve florets were harvested for the palea/lemma, the stamens and the carpel, while 20 florets were dissected for a total of 40 lodicules.

### RNA Extraction

Total RNA was extracted using the Qiagen Plant RNA Kit, Ambion Turbo RNA-free Kit and approximately 200ng of RNA used for cDNA synthesis with Superscript III reverse transcriptase (Invitrogen) according to the manufacturer’s instructions.

### Real-Time RT-qPCR and Co-expression Analysis

Primers were designed across the stop codon of each gene, forward in the gene and reverse in the 3′UTR (Supplementary Table 2). This is not only to avoid problems with sequence similarity between closely related genes, but also because the RNA in this position is less likely to be degraded.

RT-qPCR was performed as described by Burton et al. (2008). The quantity of the cDNA was assessed with four standard genes (*HvGAP*, *HvCyclophilin*, *HvTubulin* and *HvHSP70*) and normalised by relative threshold cycle value over the time course and floral organ samples individually using the average expression of the best matching three standard genes (*HvGAP* was excluded). All RT-qPCR was performed on three independent technical repeats with similar results. Transcript correlation analysis of the normalised expression values was done using the Pearson correlation function in MeV4.9.^8^ Hierarchical clustering analysis was performed using pheatmap package in R.^9^

### RNA *In situ* Hybridisation

Meristems were obtained as described, placed into FAA fixative solution (3.7% formaldehyde, 50% ethanol and 5% acetic acid) and vacuum infiltrated. Samples were dehydrated in an ethanol series which was subsequently swapped for d-lemonene (HistoChoice, Sigma), and finally paraffin wax (Paramat pastillated, Gurr) at 60°C. Embedded samples were cut into 6–8μm sections on a Leica RM2265 microtome and placed on lysine coated slides.

Dioxigenin labelled probes were made, in sense and antisense configuration, using the DIG labelling kit (Roche Diagnostics), following the manufacturer’s instructions. Primers used to generate the probes are listed in Supplementary Table 3.

Slides were dewaxed in d-lemonene and rehydrated in an ethanol series (2× 100%, 95% ethanol and 85 and 75% ethanol with 0.85% NaCl). The following steps were performed with an InsituPro robot (Invatis): Finalise rehydration, proteinase K digestion and re-dehydration. Re-dehydration was finalised with a reverse of the rehydration steps above, and the slides dried at 37°C. The following steps were also performed with the InsituPro robot: hybridisation, stringent washes, RNAse digestion, immunolabelling (AntiDIG-APconjugate, Roche) and washing. Substrate (NBT/BCIP, Roche) was added according to the manufacturer’s instructions and incubated overnight in the dark. Slides were fixed with ImmunoHistoMount (Sigma-Aldrich) and observed with a Nikon Ni-E optical microscope. Pictures were processed for colour, brightness and contrast in GIMP2.10.2 (www.gimp.org).

### Available Public Expression Data Analysis

Transcript data for barley early inflorescence development by RNA-seq were collected from supplemental data set 3 (Digel et al., 2015), selecting only the introgression line (S42-IL017) inflorescence samples grown in long day conditions.

Transcript data for rice early inflorescence meristem types were collected from supplemental data S1 (Harrop et al., 2016), selecting the MADS-box genes by name search.

Transcript data for wheat inflorescence development were collected from Supplementary Table 4 ‘List of wheat homologues similar to rice MADS-box genes’ (Feng et al., 2017).

## Results

### Identification and Phylogenetic Analysis of MIKCc MADS-Box Genes in the Barley Genome

The recent barley genome assembly contains 32 MIKCc MADS-box genes annotated as expressed sequences and a pseudogene strongly resembling *HvMADS14* with 97% identity, but only covering the latter 67% (Mascher et al., 2017; Monat et al., 2019). Through comparison with homologous genes from rice, and a tBLASTn search for available transcript sequences of barley, two genes labelled as *HvMADS27* (HORVU1Hr1G008290.1 and HORVU1HR1G008300.3) were found to be more closely related to *OsMADS23*, while another two genes, *HvMADS50* and *HvMADS33*, were identified as transcript sequences for barley, although not present in the MOREX v1 genome assembly. Additionally, a more accurate exon sequence was found for nine MIKCc genes through comparison with available transcripts for *HvMADS16*, *HvMADS18*, *HvMADS55*, *HvMADS56* and *HvMADS57*, and by analysing the genomic region with FGENESH, guided by the *OsMADS25* sequence, for *HvMADS25a*/*b*/*c*/*d*. This brings the total to 34 MIKCc MADS-box genes and one pseudogene in barley (Table 1).

**Figure 1:**
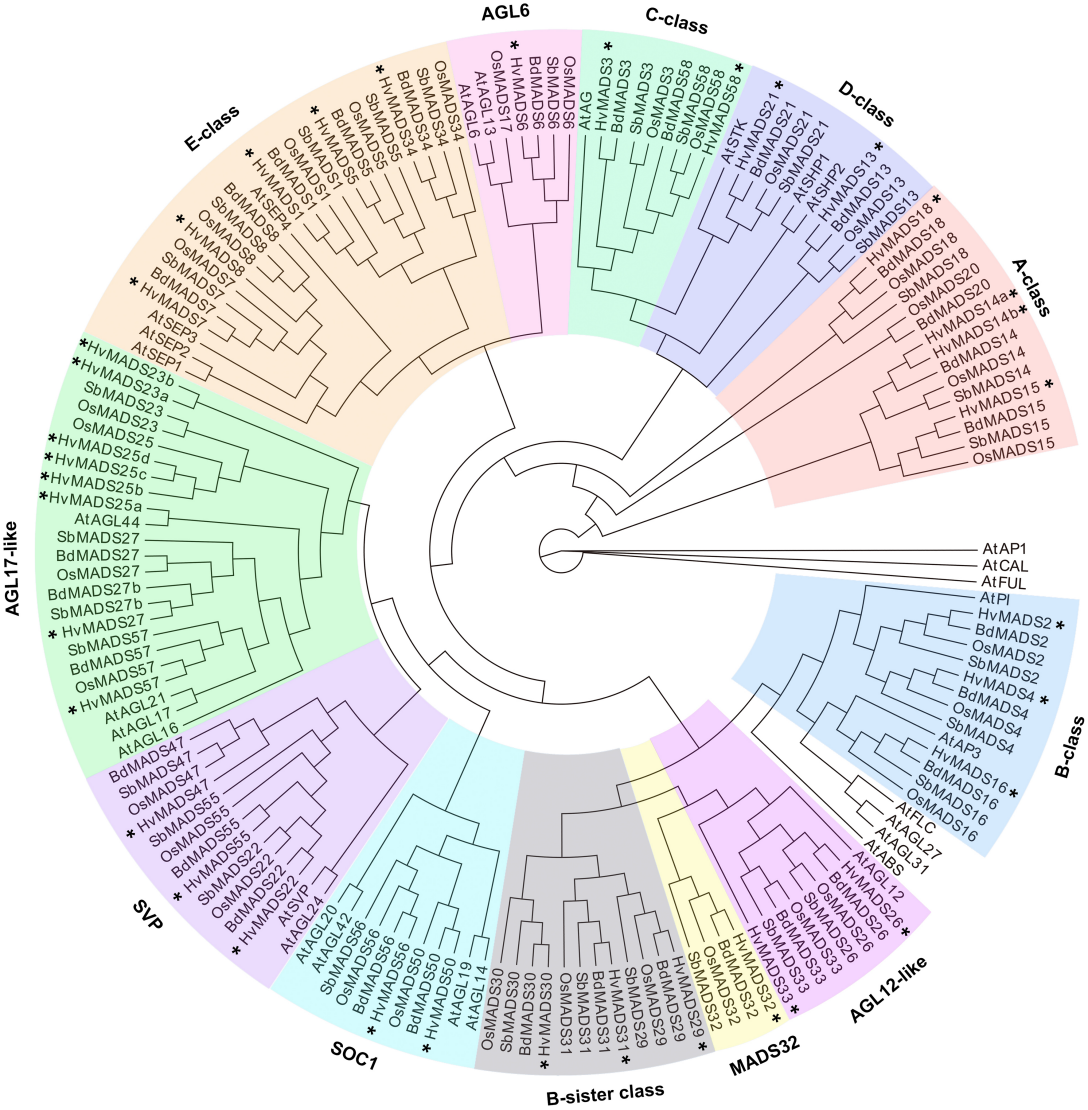
Phylogenetic analysis of all MIKCc-type MADS-box proteins (except for the truncated FLC-like proteins) in barley compared to the homologues in rice, *Brachypodium*, sorghum and *Arabidopsis*. The classes within the family are delimited by coloured boxes. SOC1, SUPRESSOR OF CONSTANS1-like proteins; SVP, SVP/VRT-like proteins. Stars (^*^) indicate the MADS-box homologues of barley.

There is a barley homologue for 31 of the 33 complete MIKCc genes in rice; missing homologues are the SQUA/AP1 gene *OsMADS20* and the AGL6-like gene *OsMADS17*. There is only one copy of *OsMADS25* in rice, but four in barley, here designated *HvMADS25a*, *HvMADS25b*, *HvMADS25c* and *HvMADS25d*. Apart from these exceptions, each barley MIKCc gene has a clear orthologue in rice. Phylogenetic analysis of encoded proteins showed that A-, B-, C-, D- and E-class proteins are conserved between rice and barley, and also with *Brachypodium* and sorghum, but show a divergence with eudicot *Arabidopsis* (Figure 1; Table 1). The FLC-like MIKCc proteins, involved in flowering time and vernalisation in *Arabidopsis* (Becker and Theißen, 2003), have truncated homologues in grasses. In barley, these homologues are *ODDSOC1* and *ODDSOC2*, which are missing the C-terminal domain and part of the keratin-like domain and fail to group with the MIKCc proteins in a phylogenetic tree based on sequence similarity alone (Ruelens et al., 2013). Conversely, the MADS32 clade has no orthologous gene group in *Arabidopsis*, or likely in any eudicot (Figure 1). Thus the overall phylogenetic analysis showed that most barley MIKCc MADS-box proteins have a close evolutionary relationship with their orthologues in rice, *Brachypodium* and sorghum, but not *Arabidopsis* (Figure 1).

The coding region of MIKCc MADS-box genes shows some expected conservation of intron-exon patterns within the different classes of closely related genes. Most exon patterns are long-short-short-medium-short-short-long, whereas intron length, and therefore gene length, varies more widely (Supplementary Figure 1). Some of the *HvMADS25* paralogous genes have big introns (a) 0.5kbp, (b) 6kbp, (c) 10kbp and (d) 15kbp.

### Low Occurrence of Single-Nucleotide Polymorphisms Shows High Conservation of MIKCc Genes’ Coding Region

To reveal the natural variation within the MIKCc MADS-box gene family during barley domestication, a comprehensive search for SNPs was performed in a database from exome sequencing of 22,626 barley cultivars, landraces and wild relatives that was recently made accessible at IPK Gatersleben, and from the barley pan-genome database that have been sequenced in 20 cultivars (Milner et al., 2019; Jayakodi et al., 2020).

The result shows that only 14 of the 34 MIKCc MADS-box genes contain any SNPs in the varieties sampled in the SNP-browser. Within these 14 genes, only half exhibit amino acid changes (Supplementary Table 1), although never in the first 110 amino acids, a region that contains the MADS domain and the Intervening domain. Remarkably, all but one of the 20 SNPs associated with *HvMADS2* occur between *HvMADS2* and its neighbouring gene HORVU.MOREX.r2.3HG0256630.1, suggesting active variation of the transcriptional interaction between these two genes. In the promoter region of *HvMADS22*, within 1kb of the start of the coding region, there are 12 SNPs of varying rarity. Three of these, 529572337T, 529572349T and 529572362A, commonly occur together and correspond to *Hybernum viborg*, a winter barley grown in many parts of the world (Supplementary Table 1). The results from pan-genome database show a different distribution of SNPs, but the amount is equally low in most of the clades. The more recently duplicated genes in the *AGL17* clade (e.g., *HvMADS25*) have more abundant SNPs, as do *HvMADS30* and *HvMADS50* to a lesser extent. Apart from four amino acid indels among all MIKCc encoded proteins and a deletion of the last six amino acids of HvMADS16, no other variety in the coding sequence was detected across the pan-genome (Supplementary Table 1).

The lack of both natural and selected variation in these genes suggests a high rate of conservation and therefore importance for fitness and domestication. In rice, there are significantly more SNPs that change an amino acid, although many occur at a very low frequency (Supplementary Table 1; Mansueto et al., 2017).^7^ These findings demonstrate that MIKCc MADS-box genes are not only conserved among grass species, but also show very few SNPs and other sequence variety between the coding sequence in both wild and domesticated barley.

### Global MIKCc Transcripts Are Concentrated in the Inflorescence and Caryopsis

**Figure 2:**
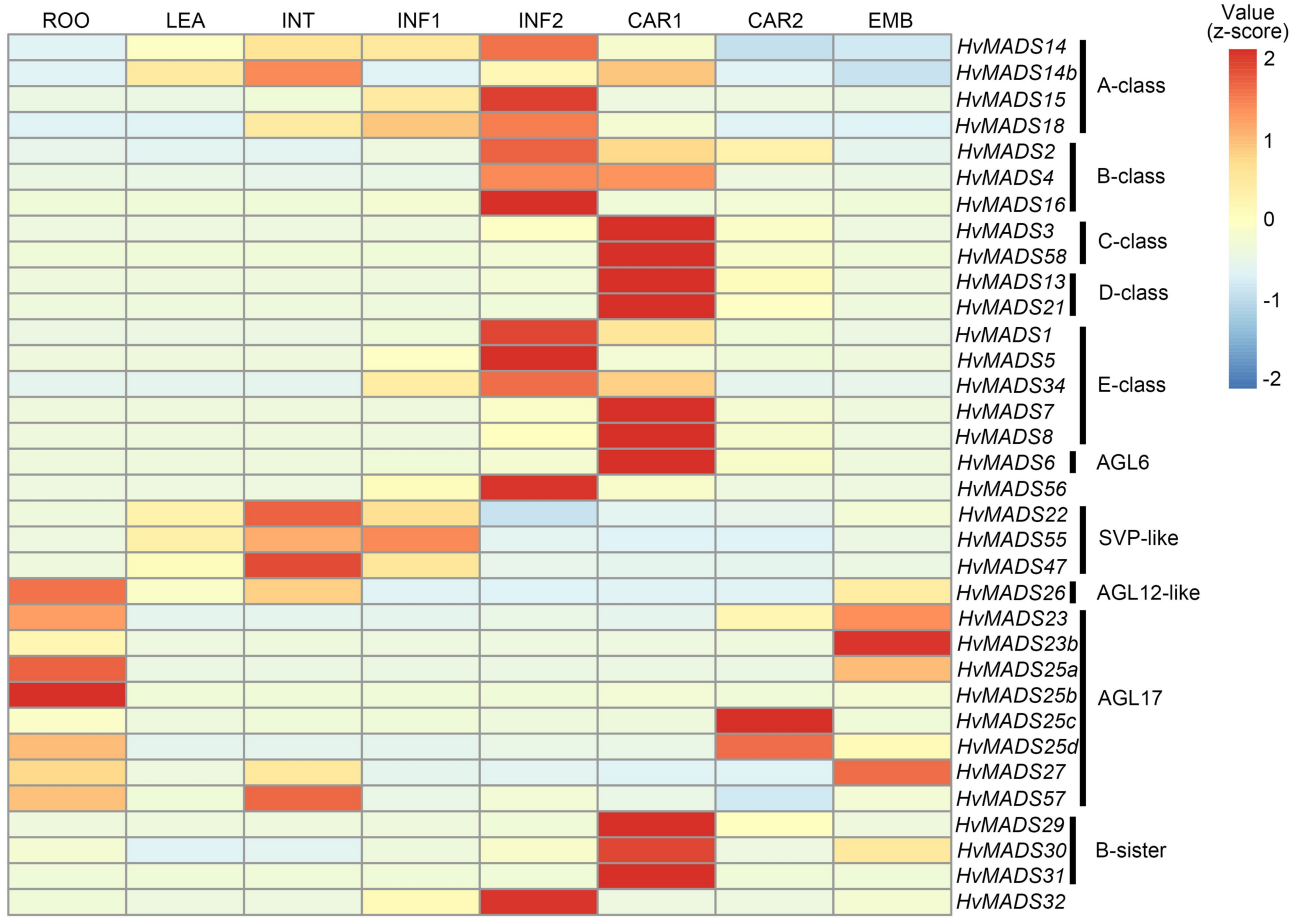
Hierarchical clustering analysis of MIKCc genes’ global transcript in different barley tissues. RPKM (Reads Per Kilobase Million) is normalised using a pheatmap package in R. ROO, roots 17days after planting (DAP); LEA, leaves 17 DAP; INT, third internode 42 DAP; INF1, developing inflorescence 5mm (about 30 DAP); INF2, developing inflorescence 10–15mm (about 50 DAP); CAR1, developing grain 5days after anthesis (DAA); CAR2, developing grain 15 DAA; and EMB, embryo 4days after germination. Raw data from Mayer et al. (2012).

The ABCDE-class MIKCc genes in barley, according to the transcript data accompanying the HORVU database (Mayer et al., 2012), are predominantly expressed in the developing inflorescence at 0.5 and 1.5cm (INF1 and INF2) and in developing seed (CAR1 and CAR2; Figure 2).

Transcript in other barley tissues, such as leaf and root, is seen for the SVP-like genes *HvMADS22/47/55*. AGL17-like genes *HvMADS23a*/*b*, *HvMADS25a*/*b*/*c*/*d* and *HvMADS27* are expressed mostly in the root and during late seed development; however, their overall transcript level is low (RPKM 10 or less), suggesting they may not be functional, or they are only expressed under stress conditions, like some AGL17-like genes in wheat (Schilling et al., 2020). *HvMADS57* has RPKMs of 25 and 35 in root and internode, respectively, which indicates it is the most likely among the AGL17-like genes to be functional. *OsMADS57* has been shown to function in cold tolerance in rice, directly targeting *OsWRKY94* and *OsD14* (Guo et al., 2013; Chen et al., 2018b).

Overall, among the 34 MIKCc MADS-box genes, most of them (20) are expressed in the developing inflorescence and, similar to homologues in related species, are probably involved in meristem transitions and floral organ development (Arora et al., 2007; Paollacci et al., 2007; Wei et al., 2014). However, to gain any insight into transcript similarities and differences of the MIKCc genes in barley inflorescence development, and to what extent the ABCDE model is likely conserved, a higher resolution transcript profile from floral transition to pollination and a complete set of floral organ transcript data would be required.

### Transcript Profiles of MIKCc Genes in Inflorescence Development and Floral Organs

**Figure 3:**
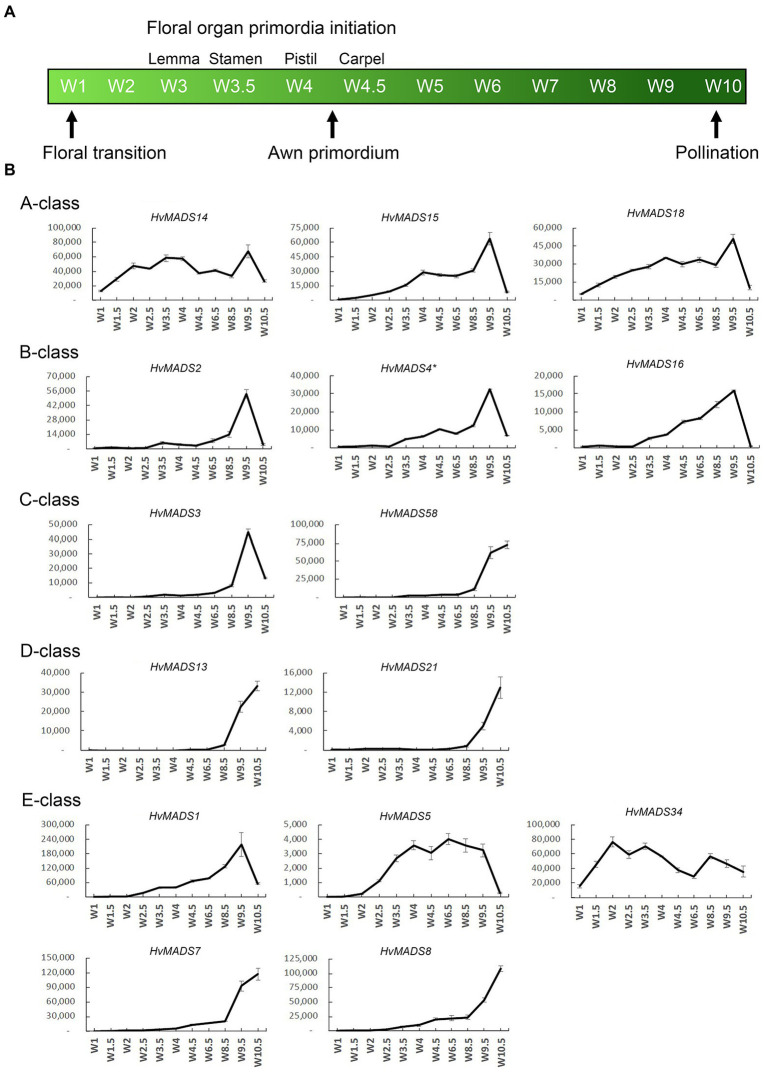
**(A)** Waddington stages for barley and their relation to major developmental steps. **(B)** Transcript profiles of AP1, AP3/PI, AG(C), AG(D) and SEP MIKCc MADS-box genes in the shoot apex through inflorescence development as measured by the Waddington stage in Golden Promise. Error bars represent one standard deviation, based on technical replicates. A-class: The predicted A-class function in the outer floral organs predicts that transcript starts after W3, where the lemma primordium is first formed. However, expression of all three AP1 genes increases earlier, at the floral transition W1. AP3/PI: The expression of predicted B-class genes starts to increase at W3.5, where the stamen primordia are formed, and peaks right before pollination. AG(C): *HvMADS3* and *HvMADS58* both start expression around W3.5 when the stamen primordia appear, however *HvMADS3* peaks before pollination and declines quickly afterwards, while HvMADS58 maintains peak expression through to W10. AG(D): *HvMADS13* and *HvMADS21* both start significant expression only after W6.5, well after the pistil primordium is formed, which first appears at W4. Their peak expression is after pollination. E-class: There is a clear difference between the LOFSEP genes *HvMADS1*, *HvMADS5* and *HvMADS34* that express earlier and sharply drop at pollination (W10) and *HvMADS7* and *HvMADS8* expression, which starts later around W3.5 and continues to rise through pollination.

**Figure 4:**
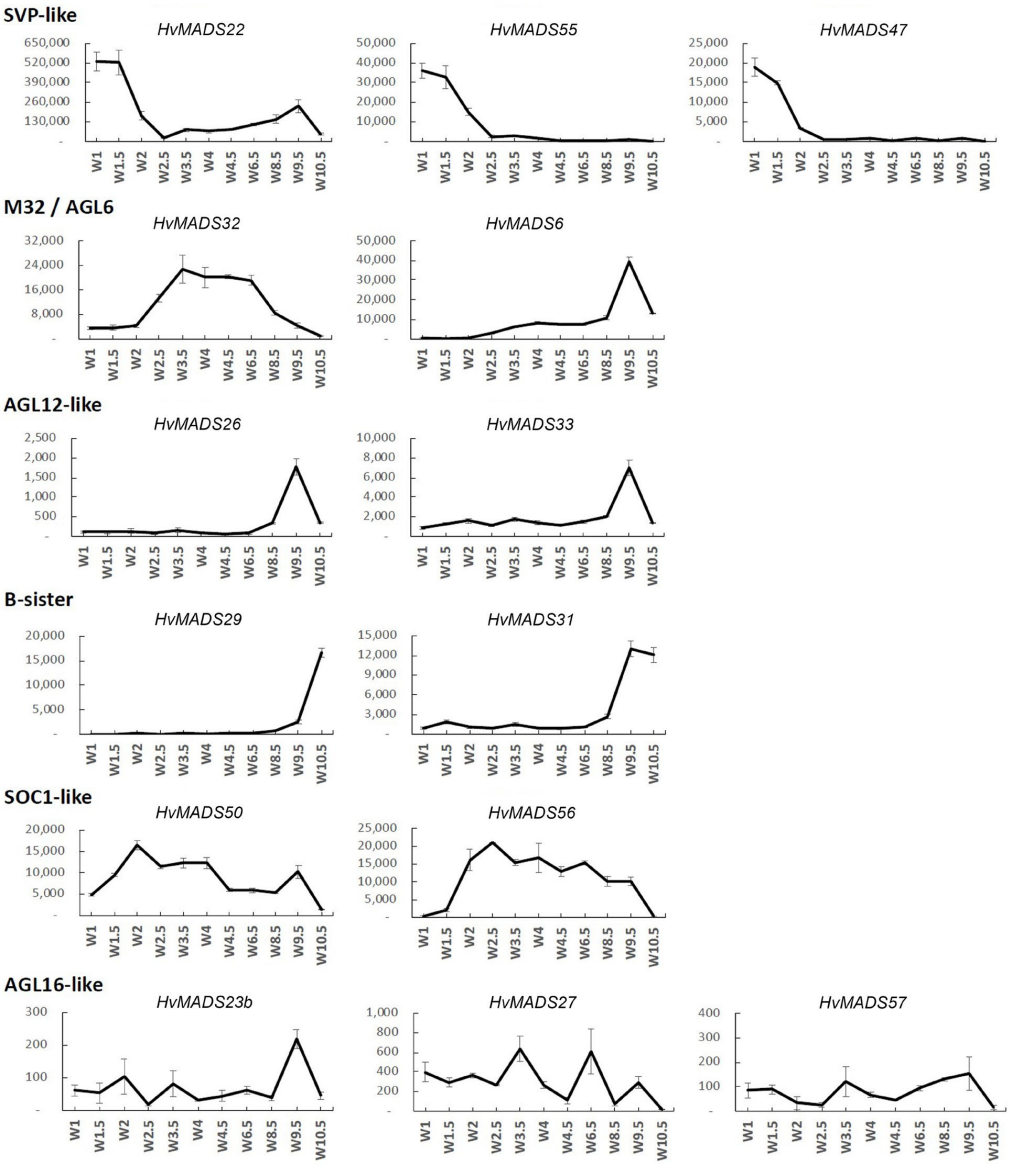
Transcript profile of the non-ABCDE MIKCc MADS-box genes through inflorescence development by Waddington stage in Golden Promise. Error bars represent one standard deviation, based on technical replicates.

**Figure 5:**
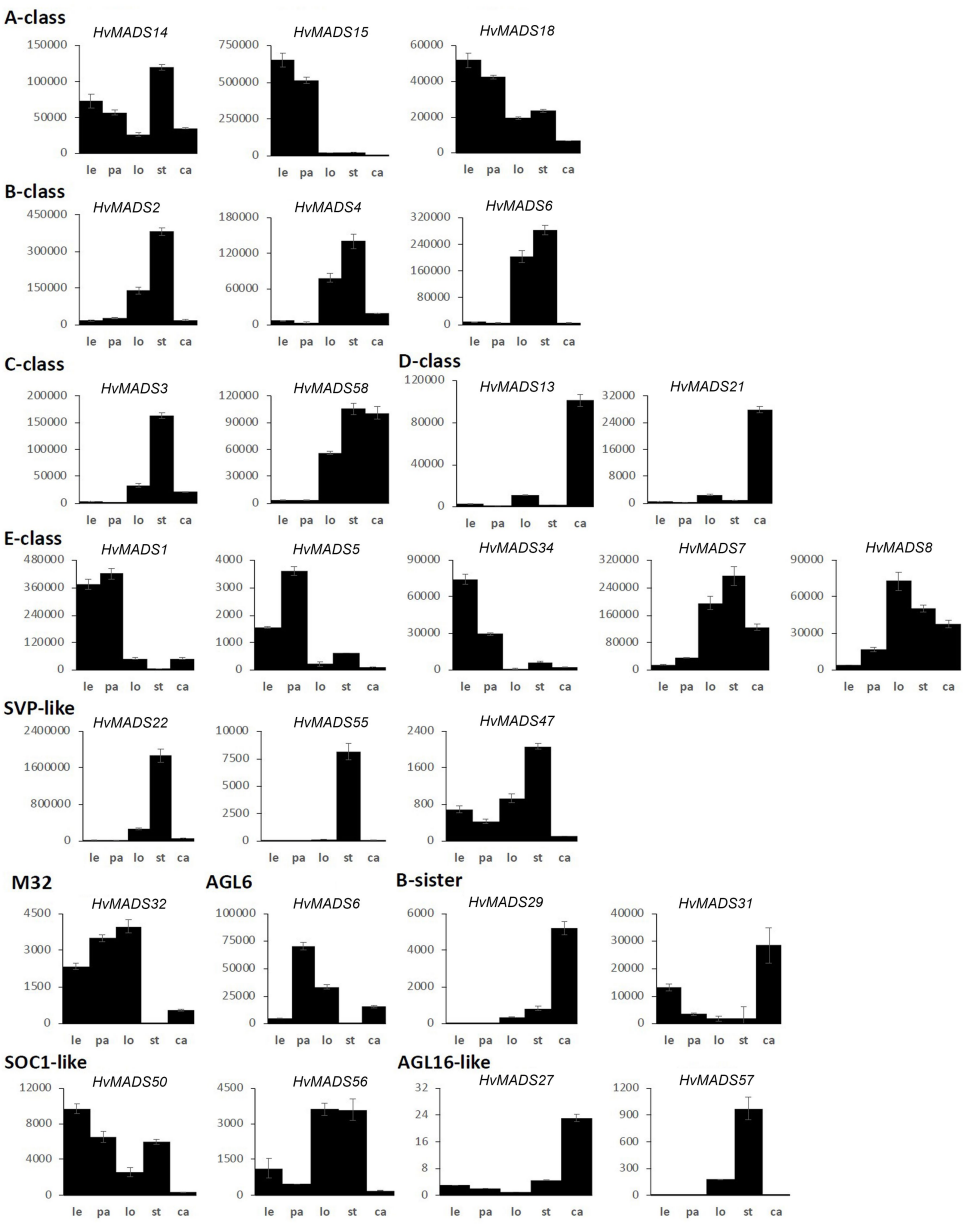
MIKCc MADS-box gene expression in floral organs at Waddington stage 9.5. Error bars represent one standard deviation, based on technical replicates. Le, lemma; pa, palea; lo, lodicules; st, stamens; and ca, carpel.

The transcript profiles of MIKCc genes through barley inflorescence development (Figures 3, 4) can be related to developmental events by Waddington stage (Figure 3A). Combined with transcript data in the floral organs at Waddington stage W9.5 (Figure 5 and Supplementary Figure 4), a comparison to established ABCDE models in other species can be made.

#### AP1 (A-Class)

Canonical A-class function in the outer floral organs would predict expression starting after the lemma primordium is first formed, at W3. However, expression of all three A-class genes increases before this, at the floral transition W1, indicating a role in earlier inflorescence development. *HvMADS14* transcript is already present at W1, peaks at W3.5, declines into W4.5 and peaks again at W9.5, right before pollination. The early expression of *HvMADS18*, along with the decline at W4.5, is less pronounced than for *HvMADS14*, but still recognisable (Figure 3B). *HvMADS15* transcript is closer to the expected profile of an A-class function gene, and transcript is indeed confined to the lemma and palea. While *HvMADS14* and *HvMADS18* are also expressed in the lemma and palea, their transcript is not confined there and *HvMADS14* is surprisingly more strongly expressed in the stamens than in the first whorl, indicating that the barley AP1 clade genes may have an additional function diverging from the classical ABCDE model (Figure 5 and Supplementary Figure 4).

#### AP3/PI (B-Class)

B-class gene transcripts start to increase at W3.5, where the stamen primordia are formed, and peak right before pollination (Figure 3B). Transcript is confined to the lodicules and stamens, exactly following the ABCDE model, indicating B-class function is likely to be completely conserved in barley (Figure 5 and Supplementary Figure 4).

#### AG (C-Class)

*HvMADS3* and *HvMADS58* both start expression around W3.5 when stamen primordia appear, in accordance with the ABCDE model. However, where *HvMADS3* peaks before pollination and declines quickly afterwards, *HvMADS58* maintains peak expression through W10, indicating subfunctionalisation of the two genes, where *HvMADS58* is responsible for the C-class function in the carpel (Figures 3, 5). Both C-class genes also show some expression in the lodicules, which does not fit with the ABCDE model (Figure 5 and Supplementary Figure 4).

#### AG (D-Class)

*HvMADS13* and *HvMADS21* both start significant expression only after W6.5, well after the pistil primordium is first formed at W4. Peak transcript is after pollination and confined to the fruit, indicating that their canonical role in ovule development and into fruit development is likely to be conserved in barley (Figure 3B).

#### SEP (E-Class)

There is a clear difference between the ‘LOFSEP’ genes *HvMADS1*, *HvMADS5* and *HvMADS34* that express earlier and sharply drop at pollination (W10) and *HvMADS7* and *HvMADS8* expression, which starts later around W3.5 and continues to rise through pollination (Figures 3A,B). Floral organ transcripts show a division along the same line, where the LOFSEP genes are mostly confined to the lemma and palea, whereas *HvMADS7* and *HvMADS8* are expressed in the lodicules, stamens and carpel (Figure 5 and Supplementary Figure 4). Therefore, the LOFSEP genes probably perform the E-class function in the lemma and palea, while *HvMADS7* and *HvMADS8* fulfil the E-class function in the other floral organs. In contrast to all other E-class genes, *HvMADS34* is expressed at W1 and peaks at W2, similar to *HvMADS14*, hinting at a function in early inflorescence development.

#### SVP-Like

The three SVP-like genes, *HvMADS22*, *HvMADS47* and *HvMADS55*, are highly expressed at the start of the floral transition and quickly decline to insignificant expression at W2.5, which indicates a role at this early stage. *HvMADS22* has a surprising resurgence in expression to a new maximum at W9.5, exclusively in the stamens, indicating possible neofunctionalisation (Figures 4, 5 and Supplementary Figure 4).

#### MADS6

*HvMADS6*, closely related to the E-class genes, has an expression profile similar to *HvMADS1* (Figure 4), but contrastingly is not expressed in the lemma, but rather in the lodicules (Figure 5 and Supplementary Figure 4). *HvMADS1* and *HvMADS6* may be partially redundant in E-class function, but not in the lemma and lodicules.

#### MADS32

*HvMADS32* has no direct equivalent in *Arabidopsis*, and no assigned function in the original ABCDE model. The *HvMADS32* transcript appears before initiation of the floral organs and uniquely declines after W6.5, unlike any other MIKCc gene (Figure 4). Floral organ expression is concentrated in the lemma, palea and lodicules (Figure 5 and Supplementary Figure 4).

#### B-Sister

*HvMADS29* and *HvMADS31* are expressed late in inflorescence development, mostly after W8.5, and are strongly expressed after pollination (Figure 4). Combined with a nearly exclusive expression in the carpel, they are likely to be involved in ovule and seed development (Figure 5 and Supplementary Figure 4).

#### SOC1-Like

*HvMADS50* and *HvMADS56* expression starts early, with a peak at W2 and W2.5, much like *HvMADS14* and *HvMADS34*. Late expression is weaker, but only disappears after pollination (Figures 4, 5 and Supplementary Figure 4).

### MIKCc Genes With Low Transcript in the Inflorescence

There was no significant transcript detected for several AGL17-like genes, including *HvMADS23a* and all four *HvMADS25* co-orthologues during the stages of inflorescence development examined here. Global expression analyses indicated that these transcripts are more prevalent in embryo, leaf and root tissue (Figure 2). Of the AGL17-like genes that did have measurable transcript, namely, *HvMADS23b*, *HvMADS27* and *HvMADS57*, low abundance and erratic profiles preclude any meaningful speculation on their function (Figure 4). The *HvMADS14b* pseudogene did seem to be expressed based on primer pair tests at various temperatures, but could never be sufficiently separated from the very similar and much more abundant *HvMADS14* transcript to provide a clear expression profile (data not shown). The final missing profile is that of *HvMADS30*, a B-sister gene, for which no expression was detected.

### Co-expression Profiles Reveal a Novel Regulatory Network Among MADs-Box Genes in Barley Developing Inflorescences

**Figure 6:**
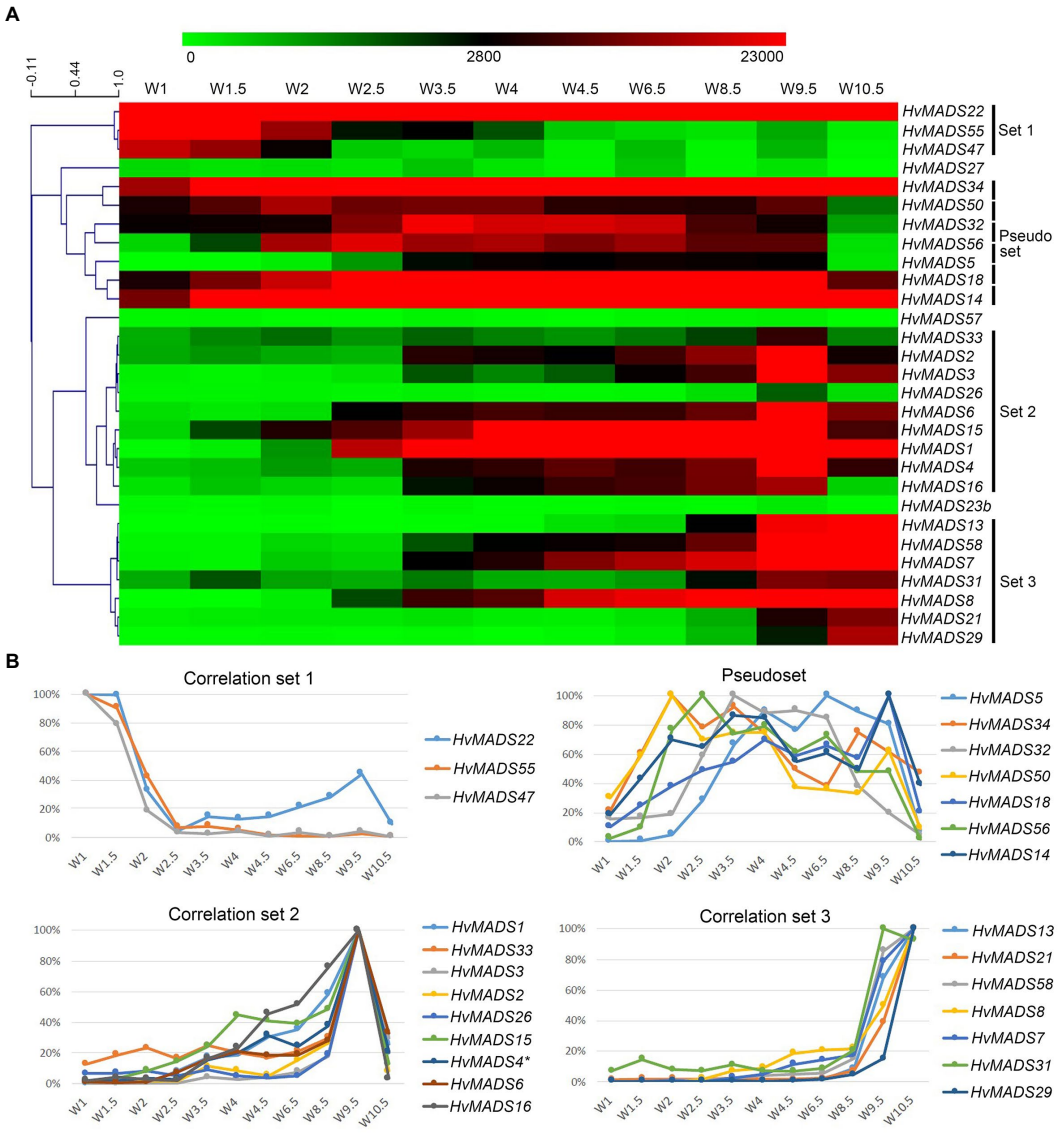
Transcript profiles of MIKCc MADS-box genes can be grouped into correlation sets. **(A)** Pearson correlation of time course expression reveals three sets and a pseudoset. Expression is represented on a logarithmic colour scale, where the maximum value is capped to provide the best visual contrast in the data set spanning orders of magnitude. Correlation tree and scale bar are presented on the left side. **(B)** Relative expression profiles through inflorescence development of the MADS-box genes within each correlation set.

To quantify co-expression of MIKCc MADS-box genes, which indicates possible functional connections, a correlation analysis was performed. Correlation analysis of the transcript profiles generated by RT-qPCR of all MIKCc MADS-box genes reveals three sets, here defined as members having a correlation coefficient of over 0.9 with at least two other members, and a less cohesive pseudoset (Figures 6A,B; Supplementary Table 4). A cluster analysis of the data showed a similar result of grouping (Supplementary Figure 5). Correlation set 1 is expressed mostly at W1 and W1.5, during the floral transition, and quickly disappears after this stage (Figure 6). In contrast, the pseudoset is spread out over the whole time course, but has some members with high transcript levels between W1.5 and W3.5 where none of the other sets show strong expression. This is the window for spikelet- and floret meristem initiation and development and then follows correlation set 2, which starts at W3 and stops after W9.5, where most floral organs develop. Finally, correlation set 3 shows transcript the latest and generally has maximum expression at W10.5, after pollination. *HvMADS23B*, *HvMADS27* and *HvMADS57* did not group into any set (Figure 6).

#### Set 1: Floral Transition

This contains three SVP-like genes, *HvMADS22*, *HvMADS47* and *HvMADS55*. Expression starts high at W1 and quickly declines to a minimum at W2.5. Remarkably, the expression of *HvMADS22* is upregulated again after W2.5 and peaks at W9.5 (Figures 4, 6). As the members of this set are already a class within the MIKCc genes, it is likely that they redundantly repress further inflorescence development.

#### Pseudoset: Expressed During Development of the Spikelet- and Floret Meristem

**Figure 7:**
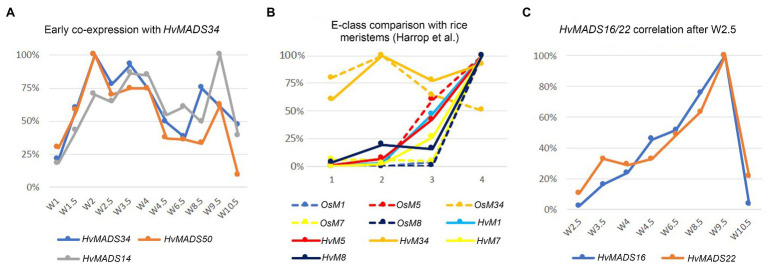
Co-expression within barley or with equivalent stages in rice can suggest related functions. **(A)** Early co-expression of *HvMADS34* with *HvMADS14* (A-class) and *HvMADS50* (SOC1-like). **(B)** Comparison of early E-class gene expression in barley and rice. Analysis of the differential gene expression in the inflorescence meristem types of rice using laser microdissection followed by RNA sequencing (dashed lines) from the supplemental data of Harrop et al. (2016). While directly comparing the results of their work with expression in the early stages of the whole barley inflorescence meristem is a false equivalency, it can still provide some insights. The best matching Waddington stages W1.5–W3.5 expression data are shown (solid lines; this paper). On the *x*-axis, 1 is inflorescence meristem/W1.5, 2 is branch meristem/W2, 3 is elongated branch meristem/W2.5 and 4 is spikelet meristem/W3.5. **(C)** Correlation of the relative expression of *HvMADS22* and *HvMADS16* between W2.5 and W10.5.

Expression of genes in the pseudoset is not as closely correlated as members of the other sets, but a general pattern can still be distinguished. Transcript mostly rises between the floral transition (W1) and emergence of the floral organs (W3–W4), and for some genes, the maximum expression is also in this early time-frame (Figure 6B). The SEP clade genes in the pseudoset are LOFSEP genes *HvMADS5* and *HvMADS34*. *HvMADS34* really stands out from the other E-class genes due to the very early high level of expression peaking at W2. *SUPPRESSOR OF OVEREXPRESSION OF CONSTANS* (*SOC1*)-like genes *HvMADS50* and *HvMADS56* are both expressed early in barley inflorescence development with a maximum at W2 and W2.5, respectively, and show a steep decline after W4. Early expression of pseudoset members indicates a function in floral development prior to the formation of the floral organs, such as a role in the spikelet- or floret meristem. Correlation of *HvMADS14*, *HvMADS34* and *HvMADS50* early expression suggests the possibility of related functions (Figure 7A).

#### Set 2: Lemma, Palea, Lodicule and Stamen Development

Correlation set 2 is not as uniform as sets 1 and 3. Transcripts in general appear around W3–W3.5, increase to a maximum right before anthesis at W9.5 and quickly diminish immediately after pollination at W10.5 (Figure 6B).

**Figure 8:**
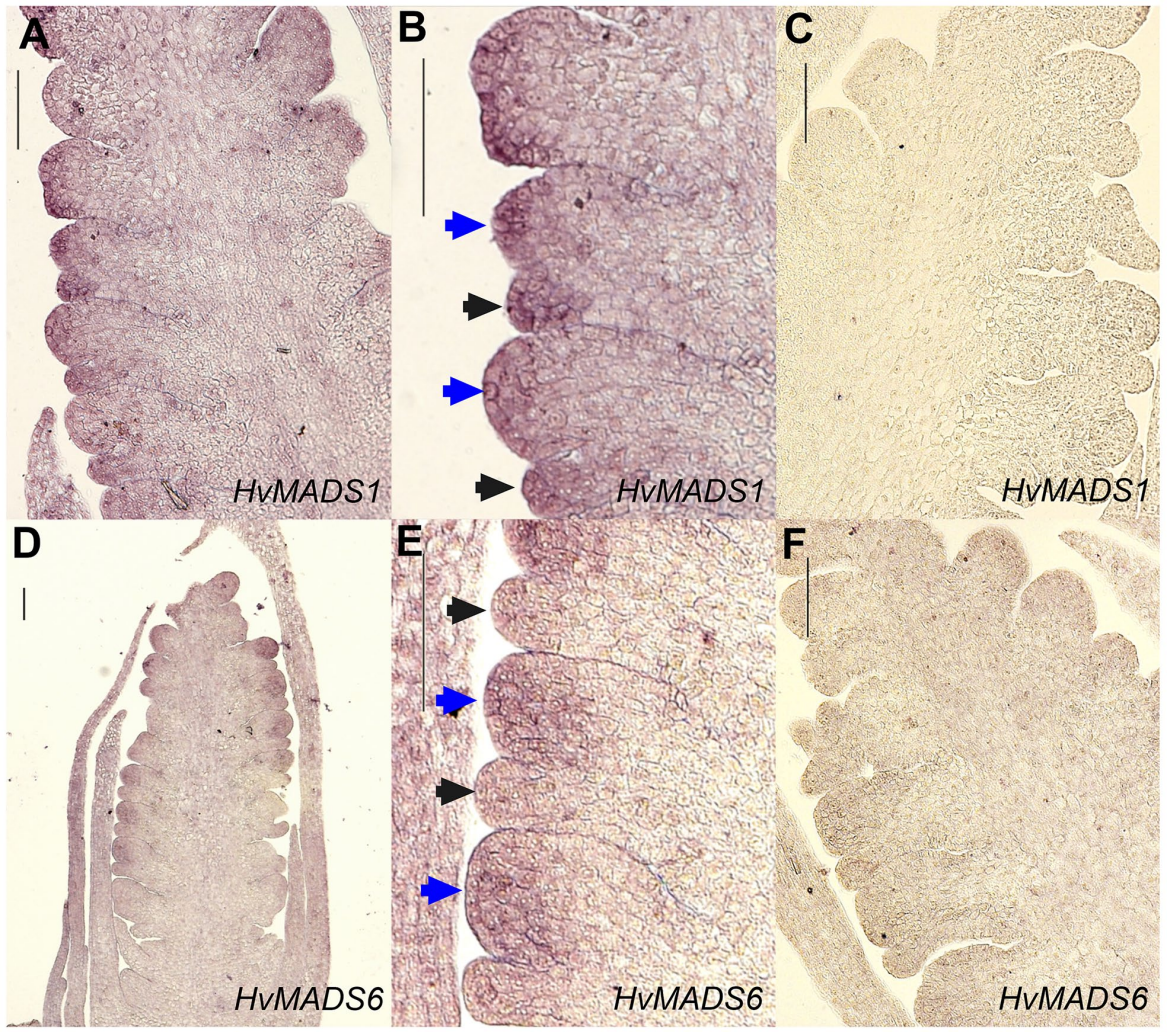
*In situ* hybridisation of *HvMADS1* and *HvMADS6* probes to sections of the barley inflorescence at W3. *HvMADS1* is expressed in the floret meristem (blue arrowheads) and the lemma primordium (black arrowheads; **A,B**), but *HvMADS6* is more strongly expressed in the floret meristem **(D,E)**. **(C, F)** are the sense probe controls. Scale bars: 100μm.

All three AP1 clade genes are strongly expressed in the lemma and palea, although the expression of *HvMADS14* and *HvMADS18* starts significantly earlier. The LOFSEP subclade, *HvMADS1*, *HvMADS5* and *HvMADS34*, are strongly expressed in the lemma and palea, but hardly at all in the lodicule, stamen and carpel (Figure 5). Of these, only *HvMADS1* appears in correlation set 2. The strongest difference in expression between the lemma and palea is for *HvMADS6*, which is expressed in the palea, but at very low levels in the lemma (Figures 5, 8). In general, this indicates A- and E-class genes are expressed in the first whorl, which is consistent with the ABCDE model in other plants.

Lodicule expression is shown for the AP1 clade genes *HvMADS14* and *HvMADS18*, all three B-class and both C-class genes *HvMADS3* and *HvMADS58* plus strongly for the SEP3 subclade of the E-class genes, *HvMADS7* and *HvMADS8*. In this tissue, the *HvMADS32* transcript profile in the pseudoset is not similar to that of set 2 members, with the most notable difference being an early sharp decline after W6.5. *HvMADS6* is also expressed in the lodicules. Canonically, the second whorl has A-, B- and E-class gene expression so the C-class transcript in barley lodicules is unexpected, and clashes strongly with the ABCDE model.

The AP1 clade genes *HvMADS14* and *HvMADS18* are both expressed in the stamens, and for *HvMADS14*, this is the highest expression seen in any floral organ. All three B-class genes and both C-class genes, *HvMADS3* and *HvMADS58* plus the E-class genes *HvMADS7* and *HvMADS8* are expressed here too, while the LOFSEP genes are only marginally expressed. Surprisingly, the highest expressed MIKCc gene in the stamens is *HvMADS22*, an SVP-like gene from correlation set 1. Ignoring the *HvMADS22* expression before W2.5, the profile thereafter is very similar to that of correlation set 2, for *HvMADS16* in particular (Figure 7C). The expected B-, C- and E-class expression for the third whorl is present in barley stamens, but the addition of A-class expression and *HvMADS22* is unexpected.

#### Set 3: Carpel and Ovule Development

Correlation set 3 contains the canonical members of a carpel and an ovule quartet: C-, D- and E-class genes. Additionally, the expression of set 3 genes peaks past pollination at W10.5. *HvMADS58*, an AG clade (C-class) gene that is part of correlation set 3, shows strong expression in the carpel, while the other AG (C-class) gene *HvMADS3*, a member of set 2, is only marginally expressed (Figure 5). The expression of D-class genes *HvMADS13* and *HvMADS21* starts late, even compared to other set 3 members, after W6.5, and is found almost exclusively in the carpel tissue. *HvMADS7* and *HvMADS8* are expressed late in floret development, and the final two genes in correlation set 3 are the B-sister genes *HvMADS29* and *HvMADS31*.

### *MADS2* and *MADS4* Are Covered by Neighbouring Kinase Transcripts

The *HvMADS4* (*HORVU1Hr1G063620*) genomic sequence is completely covered by the transcript of the neighbouring gene on the opposite DNA strand, *HORVU1Hr1G063610*, a serine/threonine protein kinase. As a result, any primer pair that targets *HvMADS4* will also amplify this protein kinase transcript. To circumvent this problem, we designed a primer pair at the end of the *HvMADS4* transcript, with an alternative reverse primer just outside of the *HvMADS4* transcript. Subtracting the signal of the latter pair from the former provides a more accurate representation of the expression level of *HvMADS4* only, which is designated HvMADS4* here.

A similar problem occurs with *HvMADS2* where the genomic span of the gene is also transcribed from the opposite direction, encoding a neighbouring kinase, *HORVU3Hr1G090990*. In this case however, the kinase expression was so low compared to the *HvMADS2* expression that trying to subtract it did not increase accuracy significantly.

## Discussion

In this study, we revealed that the MIKCc MADS-box genes in barley are highly conserved and that their expression can be grouped in correlated sets that are linked to developmental events in the spike, spikelet and floret. This suggests that floral organogenesis is regulated by the ABCDE model in barley. Phylogeny shows that the MIKCc MADS-box family in barley is highly conserved, and SNP data confirm that natural variations of MIKCc MADS-box genes do not occur frequently during barley domestication. The consistency in the number of genes in each class and the mostly one to one matching homology of MIKCc genes between barley, rice, sorghum and *Brachypodium* (Figure 1; Arora et al., 2007; Wei et al., 2014) suggests that the determination of floral organs, a process dominated by the MIKCc family, is probably conserved as well. In the genome of bread wheat, 195 MIKCc MADS-box genes have been identified (Schilling et al., 2020). This high number is not only due to hexaploidy and subsequent frequent retention of MIKCc genes, but also due to recent duplication events, theorised to help wheat adapt to diverse growth conditions. The redundancy and neofunctionalisation have led to much more frequent and severe changes in nucleotide sequences of MIKCc genes in wheat. Nevertheless, retention of functional homologues of each rice MIKCc gene suggests the conservation of their role in the inflorescence development of wheat (Schilling et al., 2020). This is also reflected in the similar floral organ development pathway seen among grasses. The ABCDE model has been extrapolated to rice, maize and wheat previously (Ciaffi et al., 2011), and the morphological and genetic conservation suggests that it may apply to barley as well. While the barley MIKCc genes are highly conserved, variation in their expression profiles can provide an insight into the robustness of the ABCDE model in barley.

Grouping MIKCc MADS-box genes using a temporal expression profile in the developing inflorescence and floral organs has its drawbacks. Many shoot apical meristem samples contain spikelets and florets at multiple stages, with younger meristems near the top (Supplementary Figure 3). Additionally, many of the ABCDE proteins are predicted to participate in multiple floral quartets, or even have roles before the floral organs are initiated, like some A-class and E-class genes, which also complicates deconvoluting transcript profiles. However, these expression profiles can still be divided into four distinct groups mathematically (Figures 6A,B; Supplementary Figure 5). The profile of each gene, combined with expression data from individual floral organs, gives a clear indication of whether each gene conforms to their expected role within the ABCDE model, as found for homologues in rice, wheat and other plant species. Nonconforming transcript profiles are also highlighted which may hint at subfunctionalisation, neofunctionalisation or new interactions that warrant further investigation. Our data showed that most barley MIKCc MADS-box genes are expressed at the specific developmental stage and in the predicted floral organs during barley inflorescence development. However, there are several strong deviations in the expression patterns of some genes expected to have an ABCDE-class function, indicating neofunctionalisation.

### SVP-Like MIKCc Genes Likely Act as Floral Inhibitors

The high start and quick decline of *HvMADS22*, *HvMADS47* and *HvMADS55* expression is in accordance with previous RNA sequencing of barley early inflorescence meristems (Digel et al., 2015; Supplementary Figure 2A) and is similar to RT-qPCR results reported by (Trevaskis et al., 2007b). In rice, the same pattern can be observed through the progression of meristem types, where *OsMADS22* and *OsMADS55* expression is high in the inflorescence meristem, lower in the branch meristem and at a minimum in the spikelet meristem (Harrop et al., 2016). In the inflorescence of *Setaria viridis*, a member of the Panicoideae (e.g. maize and sorghum) again the same decline in early inflorescence development is observed (Zhu et al., 2018). This conserved expression pattern likely indicates a conserved function of the SVP clade in grasses. In barley, the expression of *HvMADS22* peaks again at W9.5, but this re-emergence later in floret development is mirrored only in wheat (Feng et al., 2017; Supplementary Figure 2B). *HvMADS22* (*BM10*) and *HvMADS47* (*BM1*) act as floral inhibitors and can cause partial or full floral reversion when ectopically expressed (Trevaskis et al., 2007b). The expression profiles of *HvMADS22*, *HvMADS47* and *HvMADS55* fit the function as floral inhibitors well, except for the resurgence of *HvMADS22* expression.

### *APETALA* and *LOFSEP* Transcripts Dominate in the Lemma and Palea

The ABCDE model states the first floral whorl is defined by A- and E-class genes (Theissen and Saedler, 2001). However, whether the palea and lemma are true first whorl floral organs in grasses is still debated (Ciaffi et al., 2011). *HvMADS14*, *HvMADS15*, and *HvMADS18* are all strongly expressed in the lemma and palea, similar to observations in *Brachypodium* (Wei et al., 2014) and wheat (Paollacci et al., 2007), giving each APETALA gene the potential to fill the A-class role. Since *HvMADS7* and *HvMADS8* are not expressed in the lemma and palea, the E-class role is likely performed by the *LOFSEP* genes, also seen in *Brachypodium* (except for the *MADS7* homologue being expressed in the palea; Wei et al., 2014) and in wheat (Paollacci et al., 2007). The quadruple knockdown of *OsMADS1/5/7/8* (leaving *OsMADS34* as the only remaining E-class gene) transforms all floral organs in the rice floret into leaf-like structures, except for the lemma (Cui et al., 2010). As *HvMADS34* is more strongly expressed in the lemma in barley, this may be the only floral organ where HvMADS34 acts in an E-class role. Accumulation of barley *HvMADS1* mRNA also showed a high level in lemma (Figures 8A–C). Expression of *HvMADS32*, the only member of a MIKCc class unique to monocots, in the lemma and palea makes it likely to be a member of the lemma and palea floral quartet, although its unique expression profile does not match the other likely members of the quartet. Additionally, the *MADS32* homologue in *Brachypodium* shows only weak expression in the palea and not the lemma (Wei et al., 2014). The strongest difference in expression between the lemma and palea is for *HvMADS6*, which is weakly expressed in the lemma (Figures 8D–F). Even though *HvMADS6* does not belong to the SEPALLATA clade, it has been reported to fulfil an E-class function in plants (reviewed by Dreni and Zhang, 2016).

**Figure 9:**
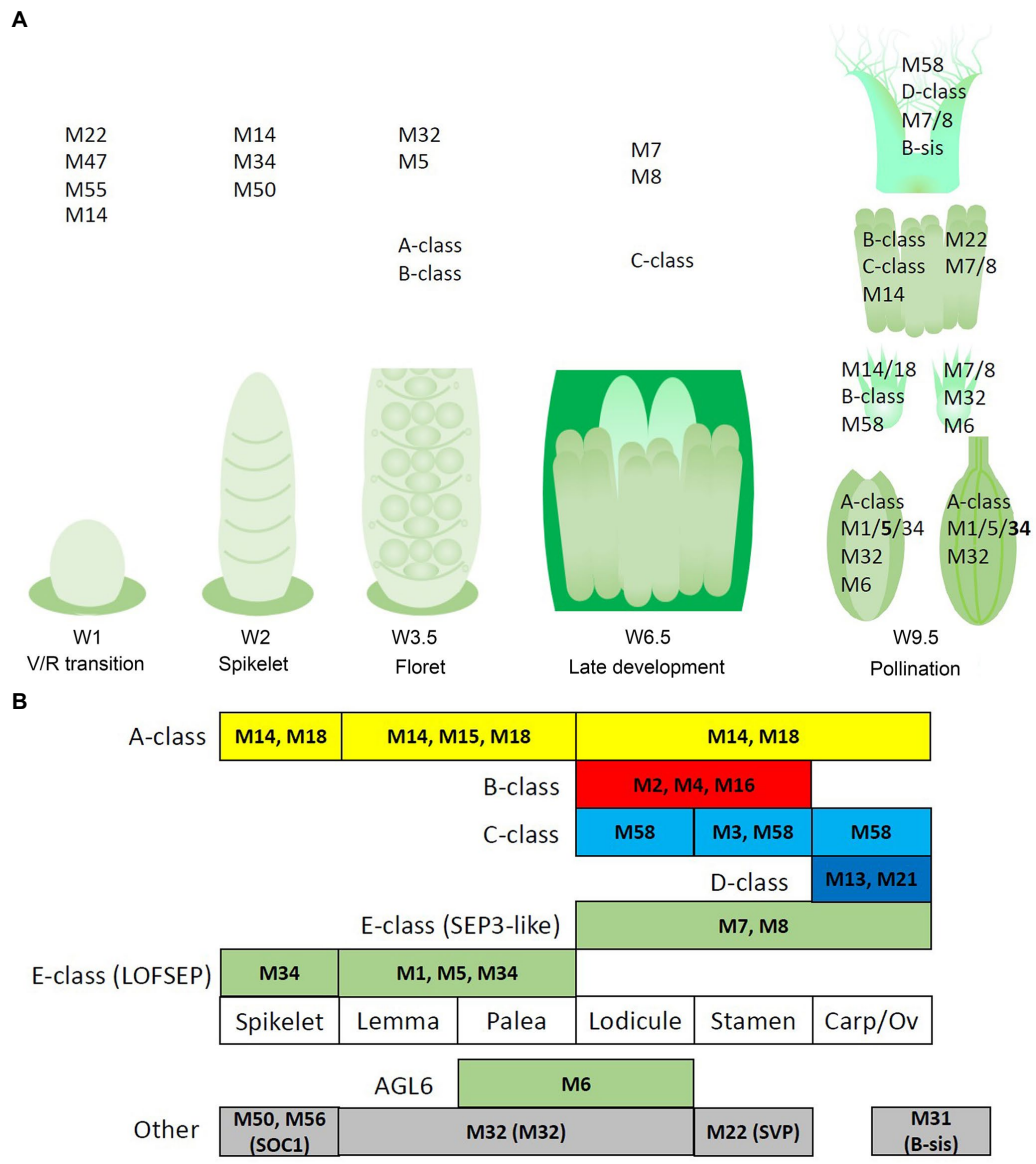
Potential regulatory networks of MIKCc MADS-box genes in barley inflorescence and floral development. In this figure, ‘MADS’ is abbreviated as ‘M’. **(A)** Prominent expression of the MIKCc MADS-box genes through developmental stages and the floral organs of barley. **(B)** Schematic of an adapted ABCDE model for barley floral development. Canonical ABCDE genes are depicted above the floral organs where they are expressed, while additional expressed MIKCc genes are shown below. Transcript before the start of floral organ primordia initiation is given in the leftmost column, tentatively labelled ‘spikelet’.

The lemma and palea floral quartets in barley are probably composed of APETALA and LOFSEP proteins, in accordance with predictions from the ABCDE model (Figure 9). In the palea, *HvMADS6* may play an E-class role in the floral quartet, possibly resulting in the morphological differences between the lemma and palea in barley.

### Lodicules Contain Predicted AP1, AP3/PI and SEP Transcript but Surprising AG (C-Class) Expression

The second whorl is canonically determined by a floral quartet consisting of one A-class, two B-class and one E-class protein. Unlike the APETALA gene *MADS15* in rice and wheat (Kyozuka et al., 2000; Paollacci et al., 2007), the barley orthologue, *HvMADS15*, is not expressed in the lodicules. All three B-class genes are expressed in the barley lodicules, in accordance with the ABCDE model and this expression is neatly matched in *Brachypodium* and wheat (Paollacci et al., 2007; Wei et al., 2014). The conservation of this B-class function and mechanism in grasses is shown by the homeotic conversion of lodicules into whorl 1-like bracts in mutants of AP3/DEF subclade members *OsMADS16*/*SUPERWOMAN1* in rice (Nagasawa et al., 2003) and *SILKY* in maize (Ambrose et al., 2000). The role of *OsMADS2* and *OsMADS4* as redundant PI/GLO seems clear from the spw1-like phenotype (homeotic conversion of stamens to carpels) of the double knockout line (Yao et al., 2008; Ciaffi et al., 2011). Moreover, the strong expression of E-class genes *HvMADS7*, *HvMADS8* and AGL6-like gene *HvMADS6* in the lodicules makes them the likely candidates for the E-class role in the lodicule-defining floral quartet in barley, which is consistent with rice homologues and maize *MADS6* homologue *ZAG3* (Thompson et al., 2009). Furthermore, the osmads7/8 double mutant shows aberrant lodicules (Cui et al., 2010), indicating that the LOFSEP (E-class) genes do not redundantly cover this function. Additionally, *HvMADS32* is strongly expressed in the lodicules, but its transcript profile in the pseudoset is not similar to that of other potential members of the lodicule quartet, most notably its early sharp decline after W6.5. However, in rice, the *osmads32* mutants do show some homeotic conversion of the lodicules (Sang et al., 2012), and a disrupted protein interaction with *OsMADS2* and *OsMADS4* is likely to be responsible for at least part of the *OsMADS32* function (Wang et al., 2015). Therefore, *HvMADS32* could have a function in lodicule determination and potentially be part of a lodicule quartet, but only in the early stages (Thompson et al., 2009).

Surprisingly, both barley C-class genes *HvMADS3* and *HvMADS58* are expressed in the lodicules, similar to homologues of wheat TaAG-1 and TaAG-2 and *Brachypodium BdMADS18*, but not with maize orthologues (*ZAG1*, *ZmM2*, and *ZmM23*; Mena et al., 1996; Paollacci et al., 2007; Wei et al., 2014). One of the central regulatory mechanisms in the ABCDE model is the antagonistic role of A- and C-class genes. In rice, the C-class genes have a role in suppressing additional lodicule formation (Yamaguchi et al., 2006). The separation of expression domains of the A-class and C-class genes, by mutual negative regulation, is one of the core tenets of the ABC model as originally devised in *Arabidopsis*. Here, we show that in barley, APETALA clade genes are expressed in the inner floral organs, and AGAMOUS clade transcripts show up in the second whorl floral organs, the lodicules.

### Stamens Contain Transcripts of Both Predicted and Unexpected Members

The A-class genes *HvMADS14* and *HvMADS18* are both expressed in the stamens, where for *HvMADS14*, it is the highest expression in any floral organ. Similarly, the wheat homologue *TaAP1-1* is expressed in all floral organs, and in *Brachypodium*, the *AP1* gene *BdMADS3* is also expressed in the stamens (Paollacci et al., 2007). In rice, *OsMADS14* and *OsMADS18* are expressed in the stamens, but *OsMADS14* is the main actor in stamen identity (Wu et al., 2017). All three B-class genes are expressed as expected, which is similar to rice, where a knockdown of the rice B-class gene *OsMADS16* or both *OsMADS2* and *OsMADS4* results in homeotic conversion of the stamens into carpel-like organs (Yao et al., 2008). C-class genes, *HvMADS3* and *HvMADS58*, are strongly expressed here, similar to their counterparts in rice. Rice *OsMADS3* plays a crucial role in stamen identity (Yamaguchi et al., 2006), but the relative importance of C-class genes in barley will have to be investigated further. Furthermore, transcripts of E-class genes *HvMADS7* and *HvMADS8* are present, where the LOFSEP genes are only marginally expressed. The homologous genes in wheat, *TaSEP4* and *TaSEP3*, are also predominantly expressed in the inner floral organs (Paollacci et al., 2007). In rice, *OsMADS7/8* double knockdown plants the stamens were affected, but not completely abolished as in the *OsMADS1/5/7/8* quadruple knockdown lines, so *OsMADS7* and *OsMADS8* have a primary E-class function, but not an exclusive one (Cui et al., 2010). Surprisingly, the most highly expressed MIKCc gene in the stamens is HvMADS22, an SVP-like gene, which normally functions as a floral repressor and in *Brachypodium* the *HvMADS22* homologue *BdMADS30* is also strongly expressed (Wei et al., 2014). Investigating expression and phenotypic differences correlated with the SNPs variations may provide insight in the *HvMADS22* role.

In summary, the canonical members of the third whorl floral quartet are expressed in barley stamens: *HvMADS16* (AP3/DEF B-class), *HvMADS2* or *HvMADS4* (PI/GLO B-class), *HvMADS3* or *HvMADS58* (C-class) and HvMADS7 or HvMADS8 (E-class). However, the APETALA clade genes *HvMADS14* and *HvMADS18*, and an SVP-like gene, *HvMADS22*, also show significant expression (Figure 9A). While the expression profiles for *HvMADS14* and *HvMADS18* do not reveal meaningful co-expression, *HvMADS22* expression after W2.5 is very similar to other probable stamen quartet members (Figure 7C), suggesting the unlikely neofunctionalisation of a floral repressor in this organ.

### *AG*, *SEP*, and B-Sister Genes Are Expressed in the Carpel and Ovule

Carpel fate is induced by a quartet of two C- and two E-class genes, while the ovule quartet contains one C-, two D- and one E-class gene (Theissen et al., 2016). Floral meristem determinacy (FMD) is likely to be regulated by the remnant of the floret meristem, located within the carpel samples.

Strong carpel expression of the AG (C-class) gene *HvMADS58*, in contrast to marginal *HvMADS3* (Figure 3B) expression, is a sign of subfunctionalisation among the *AG* genes where the C-class role in carpel development is fulfilled primarily by *HvMADS58*. The wheat homologue of *HvMADS3*, *TaAG-2*, is also predominantly expressed in the stamens, compared to the carpel (Paollacci et al., 2007). An osmads58 mutant in rice develops abnormal carpels, while osmads3 carpels develop almost completely normally, showing that *OsMADS58* is the primary C-class gene for carpel development (Yamaguchi et al., 2006). The late expression of D-class genes *HvMADS13* and *HvMADS21* is found almost exclusively in the carpel samples (collected at W9.5), which is consistent with a potential role in ovule development. *HvMADS7* and *HvMADS8*, E-class genes of the SEP3 sub-clade, are expressed late in floret development, as in wheat (Feng et al., 2017) and potentially fulfil the E-class role for the inner floral organs. For *HvMADS8* (*BM9*), this boundary at the lodicule has also been shown by *in situ* hybridisation; however, it also shows *HvMADS1* (*BM7*) expression in the developing ovule (Schmitz et al., 2000). While *HvMADS1* is expressed in the carpel at W9.5, most of it disappears after pollination. This leads to the conclusion that HvMADS7 and HvMADS8 are likely part of the carpel and ovule quartets, and *HvMADS1* may have a function during ovule development or FMD. The final two genes in correlation set 3 are the B-sister genes *HvMADS29* and *HvMADS31*. *AtABS*, a B-sister gene in *Arabidopsis*, has been linked to endothelium development and interaction with *AtSEP3*, and D-class genes suggest they may function in an additional floral quartet (Kaufmann et al., 2005). No expression of the B-sister gene *HvMADS30* was detected. *OsMADS30* is not a suitable guide for the function of its homologue in barley because the rice gene has a recent insertion and an altered expression pattern compared to related grass species (Schilling et al., 2015). However, it was recently revealed that MADS30-like gene expression is induced by biotic stress in wheat (Schilling et al., 2020), suggesting that a similar mechanism in barley may be why no *HvMADS30* expression was identified. Short genes can be expressed more rapidly than long genes and can be associated with fast dividing cells, particularly in zygotic tissue (Heyn et al., 2015). B-sister genes have short introns (Supplementary Figure 1), and their expression is associated with tissues of the ovule and developing grain. Long genes take longer to express, causing a so-called ‘intron delay’ that can be of regulatory significance. Additionally, longer genes with sizable introns are often more highly expressed (Heyn et al., 2015). However, there does not seem to be any clear correlation between intron size and frequency of expression for MIKCc genes in barley.

To summarise, the canonical MIKCc members of the fourth whorl quartet are present in the barley carpel: C-class gene *HvMADS58* and E-class genes *HvMADS7* and *HvMADS8*. The ovule quartet is also represented in the carpel samples: *HvMADS58* as the C-class gene, both D-class genes (*HvMADS13* and *HvMADS21*) and two E-class genes: *HvMADS7* and *HvMADS8* (Figures 9A,B). The additional expression of B-sister genes in this correlation set may imply a redundant function in the carpel or the ovule-determining quartet; however, it is more likely to be related to a function in the endothelium and other ovule and early seed roles. The B-sister proteins in eudicots have been shown to interact with C-, D- and E-class proteins, and the mutant has defects in the endothelium (de Folter et al., 2006).

### MIKCc Gene Expression Implies a Role in Inflorescence-, Spikelet- and Floret Meristems

MIKCc genes play an important role in grass inflorescence architecture and spikelet differentiation (Digel et al., 2015; Liu et al., 2015; Li et al., 2021; Wang et al., 2021). In our study, SVP-like genes, *HvMADS22*, *HvMADS47* and *HvMADS55*, and one A-class gene *HvMADS14* (*VRN1*), and E-class gene *HvMADS34*, show a high transcription level at the early inflorescence development stage before spikelet differentiation, suggesting a role for these genes in inflorescence meristem maintenance and spikelet meristem identity. *APETALA1* genes in barley are likely to perform A-class functions in floral organ determination (see below); however, *HvMADS14* (*VRN1*) has an additional role in the vernalisation response and probably in establishing and maintaining inflorescence meristem identity in barley (Trevaskis et al., 2007a). The early expression of *HvMADS18*, along with the decline at W4.5, is less pronounced than for *HvMADS14*, but still recognisable, indicating a potentially weaker redundant role in establishing and maintaining inflorescence meristem identity. *HvMADS15* is part of correlation set 2 and is therefore more likely to perform an A-class function exclusively. A similar divide is present in wheat, where *MADS14* and *MADS18* co-homologues have reduced expression after W4, while *MADS15* co-homologues do not (Feng et al., 2017).

Mutants in rice show that E-class *OsMADS34* gene is involved in inflorescence branching (Gao et al., 2010; Kobayashi et al., 2010), and unsurprisingly, *OsMADS34* is highly expressed in the inflorescence branch meristem of rice (Harrop et al., 2016). However, mutation of barley *HvMADS34* does not show the change of inflorescence architecture (Li et al., 2021). When comparing E-class gene expression in the early inflorescence meristem between barley and rice, the early peak of *HvMADS34* expression is conserved (Figures 7A,B). No other E-class gene (nor *HvMADS6*) could provide redundancy for a potential *HvMADS34* function around stage W2, because their expression starts later in inflorescence development. We can only speculate that the early *HvMADS34* expression is merely a vestigial remnant from the ancestral inflorescence, which did have a branched morphology (Remizowa et al., 2013). Recently, barley E-class MADS-box protein, HvMADS1, has been reported to be responsible for maintaining an unbranched spike architecture at high temperatures; the *hvmads1* mutant shows the changed inflorescence meristem determinacy at warm temperature conditions, forming a branched inflorescence-like structure (Li et al., 2021). Thus, the *Triticeae* spikes merely suppress inflorescence branching is given credence by the branching phenotype of the *com2* (*COMPOSITUM2*) and *com1/bdi1* (*COMPOSITUM1/BRANCHED AND INDETERMINATE SPIKELET 1*) mutants in barley and the tetraploid ‘miracle wheat’ (Poursarebani et al., 2015, 2020; Shang et al., 2020) and in the loss-of-function of barley *HvMADS1* mutant under high ambient temperature (Li et al., 2021) which show that most of the components for a branching inflorescence are still present in some *Triticeae*.

The loosely correlated genes in the pseudoset display early expression in the window between floral transition and the start of floral organ formation, when the spikelet and floret meristems are formed. The sudden downturn in transcript between W4 and W4.5 that many members of the pseudoset have in common (Figure 6B) coincides with the end of formation of new spikelet meristems at the awn primordium stage (Alqudah and Schnurbusch, 2014). This indicates that members of this pseudoset could be involved in inflorescence meristem determination, including the inflorescence-, spikelet- and floret meristems.

### Adapting the ABCDE Model for Barley

Overall, the ABCDE model for grasses still follows the same basic structure as the model from *Arabidopsis*, the addition of DELLA notwithstanding (Ciaffi et al., 2011). The results presented here show that this generally holds true for MIKCc gene expression in barley as well, although there are some deviations. The ABCDE proteins are known to initiate floral organ fate, as shown by mutants with homeotic changes. However, their role is not limited to just the initial direction of floral organ primordia. ABCDE proteins have been shown to bind in an organ specific way to promoter regions of genes involved in growth and differentiation of floral organ tissues, up to *SPOROCYTELESS*, a master regulator of gametogenesis in *Arabidopsis* (Chen et al., 2018a). The rising expression levels of most ABCDE-class genes throughout floret development indicate this continuous affirmation of organ identity by floral quartets may be present in barley as well. These persistent roles make floral organ sample collection at W9.5 a reasonable predictor of floral organ fate determining ABCDE-class genes. However, when looking at the MIKCc genes outside the ABCDE-functions, the most strongly expressed genes are *HvMADS22* in the stamens, usually classified as a floral repressor, and *HvMADS32*, which may be crucial for the discrete border between the outer and inner floral organs (Figure 9B). Some basal angiosperms have a more gradual transition between their floral organs, which does not fit with the ABCDE model, which results in discrete floral whorls. This is accompanied by a more gradual change in gene expression in these taxa and is captured in the ‘fading borders’ model (Buzgo et al., 2004; Soltis et al., 2007). This states that the gradually rising expression of, for example, C-class genes, and the slowly fading expression of A-class genes, results in intermediate floral organs with some characteristics from the adjacent organs. This may be the ancestral angiosperm ABC model, where only later more stringent restrictions on the expression evolved to separate the second and third whorls, resulting in the A–C antagonism in the ABCDE model for eudicots, and perhaps a different solution evolved in grasses, involving *HvMADS32*.

Because MIKCc proteins function in floral quartets, the next step to gain more insight into the potentially changed roles of these genes should be protein interaction studies. So far, when discussing the ABCDE model, the floral organs have often been considered indivisible units that either gain the correct identity or are homeotically converted. In the barley stamens, 10 different MIKCc genes from five classes are strongly expressed (Figure 9B), which are unlikely to form just one floral quartet. There may be variants of the stamen quartet that help define specific tissues within the stamens or even complete quartets. Alternatively, some of these MIKCc genes may have a function in stamens independent of a floral quartet structure. A somewhat similar tissue-specific expression of MIKCc genes has been shown in the ovule of rice (Kubo et al., 2013).

Altogether, these findings show that while the general setup of flowering is conserved, there are many interesting deviations in barley, and likely other grasses, that merit further research into what they mean for both the evolution of flowering in grasses and the potential adaptability of the inflorescence for crop yield breeding.

## Data Availability Statement

The original contributions presented in the study are included in the article/Supplementary Material; further inquiries can be directed to the corresponding authors.

## Author Contributions

Study was conceived by HK and DZ. Experiments were done by HK, NS, and SK. Bioinformatics were done by HK, JSh, and JSc. Data analysis was done by HK, NS, and GL. Manuscript was written by HK, GL, and RB. Supervision and funding by RB and DZ. All authors contributed to the article and approved the submitted version.

## Conflict of Interest

The authors declare that the research was conducted in the absence of any commercial or financial relationships that could be construed as a potential conflict of interest.

## Publisher’s Note

All claims expressed in this article are solely those of the authors and do not necessarily represent those of their affiliated organizations, or those of the publisher, the editors and the reviewers. Any product that may be evaluated in this article, or claim that may be made by its manufacturer, is not guaranteed or endorsed by the publisher.

## Footnotes

^1^ https://webblast.ipk-gatersleben.de/barley_ibsc/downloads/

^2^ https://blast.ncbi.nlm.nih.gov/Blast.cgi

^3^ https://www.uniprot.org/

^4^https://bridge.ipk-gatersleben.de/#snpbrowser, accessed April 2019.

^5^https://barley-pangenome.ipk-gatersleben.de/, accessed February 2021.

^6^ http://ugene.net/

^7^http://snp-seek.irri.org/_snp.zul, accessed June 2019.

^8^ http://mev.tm4.org

^9^ https://github.com/raivokolde/pheatmap

## References

[ref1] Alqudah A. M. Schnurbusch T. (2014). Awn primordium to tipping is the most decisive developmental phase for spikelet survival in barley. Funct. Plant Biol. 41:424. 10.1071/FP13248, PMID: 32481002

[ref2] Alvarez-Buylla E. R. Benítez M. Corvera-Poiré A. Chaos Cador A. de Folter S. Gamboa de Buen A.. (2010). Flower development. Arabidopsis Book 8:e0127. 10.1199/tab.0127, PMID: 22303253 PMC3244948

[ref3] Ambrose B. A. Lerner D. R. Ciceri P. Padilla C. M. Yanofsky M. F. Schmidt R. J. (2000). Molecular and genetic analyses of the silky1 gene reveal conservation in floral organ specification between eudicots and monocots. Mol. Cell 5, 569–579. 10.1016/S1097-2765(00)80450-5, PMID: 10882141

[ref4] Angenent G. C. Franken J. Busscher M. Vandijken A. Vanwent J. L. Dons H. J. M.. (1995). A novel class of mads box genes is involved in ovule development in petunia. Plant Cell 7, 1569–1582. 10.1105/tpc.7.10.1569, PMID: 7580252 PMC161013

[ref5] Arora R. Agarwal P. Ray S. Singh A. K. Singh V. P. Tyagi A. K.. (2007). MADS-box gene family in rice: genome-wide identification, organization and expression profiling during reproductive development and stress. BMC Genomics 8:242. 10.1186/1471-2164-8-242, PMID: 17640358 PMC1947970

[ref6] Becker A. Theißen G. (2003). The major clades of MADS-box genes and their role in the development and evolution of flowering plants. Mol. Phylogenet. Evol. 29, 464–489. 10.1016/s1055-7903(03)00207-0, PMID: 14615187

[ref7] Bommert P. Satoh-Nagasawa N. Jackson D. Hirano H.-Y. (2005). Genetics and evolution of inflorescence and flower development in grasses. Plant Cell Physiol. 46, 69–78. 10.1093/pcp/pci504, PMID: 15659432

[ref8] Bowman J. L. Smyth D. R. (1999). CRABS CLAW, a gene that regulates carpel and nectary development in *Arabidopsis*, encodes a novel protein with zinc finger and helix-loop-helix domains. Development 126, 2387–2396. 10.1242/dev.126.11.2387, PMID: 10225998

[ref9] Bremer K. (2002). Gondwanan evolution of the grass alliance of families (Poales). Evolution 56, 1374–1387. 10.1111/j.0014-3820.2002.tb01451.x, PMID: 12206239

[ref10] Burton R. A. Jobling S. A. Harvey A. J. Shirley N. J. Mather D. E. Bacic A.. (2008). The genetics and transcriptional profiles of the cellulose synthase-like HvCslF gene family in barley. Plant Physiol. 146, 1821–1833. 10.1104/pp.107.114694, PMID: 18258691 PMC2287353

[ref11] Buzgo M. Soltis D. E. Soltis P. S. Ma H. (2004). Towards a comprehensive integration of morphological and genetic studies of floral development. Trends Plant Sci. 9, 164–173. 10.1016/j.tplants.2004.02.003, PMID: 15063866

[ref12] Callens C. Tucker M. R. Zhang D. Wilson Z. A. (2018). Dissecting the role of MADS-box genes in monocot floral development and diversity. J. Exp. Bot. 69, 2435–2459. 10.1093/jxb/ery086, PMID: 29718461

[ref13] Castelán-Muñoz N. Herrera J. Cajero-Sánchez W. Arrizubieta M. Trejo C. García-Ponce B.. (2019). MADS-box genes are key components of genetic regulatory networks involved in abiotic stress and plastic developmental responses in plants. Front. Plant Sci. 10:853. 10.3389/fpls.2019.00853, PMID: 31354752 PMC6636334

[ref14] Chen D. Yan W. Fu L. Y. Kaufmann K. (2018a). Architecture of gene regulatory networks controlling flower development in *Arabidopsis thaliana*. Nat. Commun. 9:4534. 10.1038/s41467-018-07850-2, PMID: 30382087 PMC6208445

[ref15] Chen F. Zhang X. Liu X. Zhang L. (2017). Evolutionary analysis of MIKCc-type MADS-box genes in gymnosperms and angiosperms. Front. Plant Sci. 8:895. 10.3389/fpls.2017.02248, PMID: 28611810 PMC5447709

[ref16] Chen L. Zhao Y. Xu S. Zhang Z. Xu Y. Zhang J.. (2018b). OsMADS57 together with OsTB1 coordinates transcription of its target *OsWRKY94* and *D14* to switch its organogenesis to defense for cold adaptation in rice. New Phytol. 218, 219–231. 10.1111/nph.14977, PMID: 29364524 PMC5873253

[ref17] Ciaffi M. Paolacci A. R. Tanzarella O. A. Porceddu E. (2011). Molecular aspects of flower development in grasses. Sex. Plant Reprod. 24, 247–282. 10.1007/s00497-011-0175-y, PMID: 21877128

[ref18] Coen E. S. Meyerowitz E. M. (1991). The war of the whorls – genetic interactions controlling flower development. Nature 353, 31–37. 10.1038/353031a0, PMID: 1715520

[ref19] Colombo M. Masiero S. Vanzulli S. Lardelli P. Kater M. M. Colombo L. (2008). AGL23, a type I MADS-box gene that controls female gametophyte and embryo development in *Arabidopsis*. Plant J. 54, 1037–1048. 10.1111/j.1365-313X.2008.03485.x, PMID: 18346189

[ref20] Cui R. Han J. Zhao S. Su K. Wu F. Du X.. (2010). Functional conservation and diversification of class E floral homeotic genes in rice (*Oryza sativa*). Plant J. 61, 767–781. 10.1111/j.1365-313X.2009.04101.x, PMID: 20003164

[ref21] de Folter S. Shchennikova A. V. Franken J. Busscher M. Baskar R. Grossniklaus U.. (2006). A B-sister MADS-box gene involved in ovule and seed development in petunia and *Arabidopsis*. Plant J. 47, 934–946. 10.1111/j.1365-313X.2006.02846.x, PMID: 16925602

[ref22] Digel B. Pankin A. von Korff M. (2015). Global transcriptome profiling of developing leaf and shoot apices reveals distinct genetic and environmental control of floral transition and inflorescence development in barley. Plant Cell 27, 2318–2334. 10.1105/tpc.15.00203, PMID: 26307377 PMC4815099

[ref23] Dreni L. Pilatone A. Yun D. Erreni S. Pajoro A. Caporali E.. (2011). Functional analysis of all AGAMOUS subfamily members in rice reveals their roles in reproductive organ identity determination and meristem determinacy. Plant Cell 23, 2850–2863. 10.1105/tpc.111.087007, PMID: 21810995 PMC3180796

[ref24] Dreni L. Zhang D. (2016). Flower development: the evolutionary history and functions of the AGL6 subfamily MADS-box genes. J. Exp. Bot. 67, 1625–1638. 10.1093/jxb/erw046, PMID: 26956504

[ref25] Edgar R. C. (2004). MUSCLE: multiple sequence alignment with high accuracy and high throughput. Nucleic Acids Res. 32, 1792–1797. 10.1093/nar/gkh340, PMID: 15034147 PMC390337

[ref26] Feng N. Song G. Guan J. Chen K. Jia M. Huang D.. (2017). Transcriptome profiling of wheat inflorescence development from spikelet initiation to floral patterning identified stage-specific regulatory genes. Plant Physiol. 174, 1779–1794. 10.1104/pp.17.00310, PMID: 28515146 PMC5490901

[ref27] Gao X. Liang W. Yin C. Ji S. Wang H. Su X.. (2010). The SEPALLATA-like gene OsMADS34 is required for rice inflorescence and spikelet development. Plant Physiol. 153, 728–740. 10.1104/pp.110.156711, PMID: 20395452 PMC2879775

[ref28] Guo S. Xu Y. Liu H. Mao Z. Zhang C. Ma Y.. (2013). The interaction between OsMADS57 and OsTB1 modulates rice tillering via DWARF14. Nat. Commun. 4:1566. 10.1038/ncomms2542, PMID: 23463009 PMC3615354

[ref29] Harrop T. W. Ud Din I. Gregis V. Osnato M. Jouannic S. Adam H.. (2016). Gene expression profiling of reproductive meristem types in early rice inflorescences by laser microdissection. Plant J. 86, 75–88. 10.1111/tpj.13147, PMID: 26932536

[ref001] Heyn P. Kalinka A. T. Tomancak P. Neugebauer K. M. (2015). Introns and gene expression: cellular constraints, transcriptional regulation, and evolutionary consequences. Bioessays 37, 148–154. 10.1002/bies.201400138, PMID: 25400101 PMC4654234

[ref30] Jayakodi M. Padmarasu S. Haberer G. Bonthala V. S. Gundlach H. Monat C.. (2020). The barley pan-genome reveals the hidden legacy of mutation breeding. Nature 588, 284–289. 10.1038/s41586-020-2947-8, PMID: 33239781 PMC7759462

[ref31] Jin J. Zhang H. Kong L. Gao G. Luo J. (2014). PlantTFDB 3.0: a portal for the functional and evolutionary study of plant transcription factors. Nucleic Acids Res. 42, D1182–D1187. 10.1093/nar/gkt1016, PMID: 24174544 PMC3965000

[ref32] Kaufmann K. Anfang N. Saedler H. Theissen G. (2005). Mutant analysis, protein-protein interactions and subcellular localization of the *Arabidopsis* B-sister (ABS) protein. Mol. Gen. Genomics. 274, 103–118. 10.1007/s00438-005-0010-y, PMID: 16080001

[ref33] Kobayashi K. Maekawa M. Miyao A. Hirochika H. Kyozuka J. (2010). PANICLE PHYTOMER2 (PAP2), encoding a SEPALLATA subfamily MADS-box protein, positively controls spikelet meristem identity in rice. Plant Cell Physiol. 51, 47–57. 10.1093/pcp/pcp166, PMID: 19933267 PMC2807174

[ref34] Koppolu R. Schnurbusch T. (2019). Developmental pathways for shaping spike inflorescence architecture in barley and wheat. J. Integr. Plant Biol. 61, 278–295. 10.1111/jipb.12771, PMID: 30609316

[ref35] Kubo T. Fujita M. Takahashi H. Nakazono M. Tsutsumi N. Kurata N. (2013). Transcriptome analysis of developing ovules in rice isolated by laser microdissection. Plant Cell Physiol. 54, 750–765. 10.1093/pcp/pct029, PMID: 23411663

[ref36] Kwantes M. Liebsch D. Verelst W. (2012). How MIKC* MADS-Box genes originated and evidence for their conserved function throughout the evolution of vascular plant gametophytes. Mol. Biol. Evol. 29, 293–302. 10.1093/molbev/msr200, PMID: 21813465

[ref37] Kyozuka J. Kobayashi T. Morita M. Shimamoto K. (2000). Spatially and temporally regulated expression of rice MADS box genes with similarity to *Arabidopsis* class A, B and C genes. Plant Cell Physiol. 41, 710–718. 10.1093/pcp/41.6.710, PMID: 10945340

[ref38] Li G. Kuijer H. N. J. Yang X. Liu H. Shen C. Shi J.. (2021). MADS1 maintains barley spike morphology at high ambient temperatures. Nat. Plants. 10.1038/s41477-021-00957-3, PMID: 34183784

[ref39] Liu H. Li G. Yang X. Kuijer H. N. J. Liang W. Zhang D. (2020). Transcriptome profiling reveals phase-specific gene expression in the developing barley inflorescence. Crop J. 8, 71–86. 10.1016/j.cj.2019.04.005

[ref40] Mansueto L. Fuentes R. R. Borja F. N. Detras J. Abriol-Santos J. M. Chebotarov D.. (2017). Rice SNP-seek database update: new SNPs, indels, and queries. Nucleic Acids Res. 45, D1075–D1081. 10.1093/nar/gkw1135, PMID: 27899667 PMC5210592

[ref41] Mascher M. Gundlach H. Himmelbach A. Beier S. Twardziok S. O. Wicker T.. (2017). A chromosome conformation capture ordered sequence of the barley genome. Nature 544, 427–433. 10.1038/nature22043, PMID: 28447635

[ref42] Mayer K. F. X. Waugh R. Langridge P. Close T. J. Wise R. P. Graner A.. (2012). A physical, genetic and functional sequence assembly of the barley genome. Nature 491, 711–716. 10.1038/nature11543, PMID: 23075845

[ref43] Mena M. Ambrose B. A. Meeley R. B. Briggs S. P. Yanofsky M. F. Schmidt R. J. (1996). Diversification of C-function activity in maize flower development. Science 274, 1537–1540. 10.1126/science.274.5292.1537, PMID: 8929416

[ref44] Milner S. G. Jost M. Taketa S. Mazón E. R. Himmelbach A. Oppermann M.. (2019). Genebank genomics highlights the diversity of a global barley collection. Nat. Genet. 51, 319–326. 10.1038/s41588-018-0266-x, PMID: 30420647

[ref45] Monat C. Padmarasu S. Lux T. Wicker T. Gundlach H. Himmelbach A.. (2019). TRITEX: chromosome-scale sequence assembly of Triticeae genomes with open-source tools. Genome Biol. 20:284. 10.1186/s13059-019-1899-5, PMID: 31849336 PMC6918601

[ref46] Nagasawa N. Miyoshi M. Sano Y. Satoh H. Hirano H. Sakai H.. (2003). SUPERWOMAN1 and DROOPING LEAF genes control floral organ identity in rice. Development 130, 705–718. 10.1242/dev.00294, PMID: 12506001

[ref47] Paollacci A. R. Tanzarella O. A. Porceddu E. Varotto S. Ciaffi M. (2007). Molecular and phylogenetic analysis of MADS-box genes of MIKC type and chromosome location of SEP-like genes in wheat (*Triticum aestivum* L.). Mol. Gen. Genomics. 278, 689–708. 10.1007/s00438-007-0285-2, PMID: 17846794

[ref48] Pelaz S. Ditta G. S. Baumann E. Wisman E. Yanofsky M. F. (2000). B and C floral organ identity functions require SEPALLATA MADS-box genes. Nature 405, 200–203. 10.1038/35012103, PMID: 10821278

[ref49] Poursarebani N. Seidensticker T. Koppolu R. Trautewig C. Gawronski P. Bini F.. (2015). The genetic basis of composite spike form in barley and “miracle-wheat”. Genetics 201, 155–165. 10.1534/genetics.115.176628, PMID: 26156223 PMC4566260

[ref50] Poursarebani N. Trautewig C. Melzer M. Nussbaumer T. Lundqvist U. Rutten T.. (2020). COMPOSITUM 1 contributes to the architectural simplification of barley inflorescence via meristem identity signals. Nat. Commun. 11:5138. 10.1038/s41467-020-18890-y, PMID: 33046693 PMC7550572

[ref51] Remizowa M. V. Rudall P. J. Choob V. V. Sokoloff D. D. (2013). Racemose inflorescences of monocots: structural and morphogenetic interaction at the flower/inflorescence level. Ann. Bot. 112, 1553–1566. 10.1093/aob/mcs246, PMID: 23172413 PMC3828938

[ref52] Ruelens P. de Maagd R. A. Proost S. Theißen G. Geuten K. Kaufmann K. (2013). FLOWERING LOCUS C in monocots and the tandem origin of angiosperm-specific MADS-box genes. Nat. Commun. 4:2280. 10.1038/ncomms3280, PMID: 23955420

[ref53] Sang X. C. Li Y. F. Luo Z. K. Ren D. Y. Fang L. K. Wang N.. (2012). Chimeric floral Organs1, encoding a monocot-specific MADS box protein, regulates floral organ identity in rice. Plant Physiol. 160, 788–807. 10.1104/pp.112.200980, PMID: 22891238 PMC3461556

[ref54] Schilling S. Gramzow L. Lobbes D. Kirbis A. Weilandt L. Hoffmeier A.. (2015). Non-canonical structure, function and phylogeny of the B-sister MADS-box gene OsMADS30 of rice (*Oryza sativa*). Plant J. 84, 1059–1072. 10.1111/tpj.13055, PMID: 26473514

[ref55] Schilling S. Kennedy A. Pan S. Jermiin L. S. Melzer R. (2020). Genome-wide analysis of MIKC-type MADS-box genes in wheat: pervasive duplications, functional conservation and putative neofunctionalization. New Phytol. 225, 511–529. 10.1111/nph.16122, PMID: 31418861

[ref56] Schmitz J. Franzen R. Ngyuen T. H. Garcia-Maroto F. Pozzi C. Salamini F.. (2000). Cloning, mapping and expression analysis of barley MADS-box genes. Plant Mol. Biol. 42, 899–913. 10.1023/A:1006425619953, PMID: 10890536

[ref57] Shang Y. Yuan L. Di Z. Jia Y. Zhang Z. Li S.. (2020). A CYC/TB1-type TCP transcription factor controls spikelet meristem identity in barley. J. Exp. Bot. 71–22, 7118–7131. 10.1093/jxb/eraa416, PMID: 32915968

[ref58] Smaczniak C. Immink R. G. H. Angenent G. C. Kaufmann K. (2012). Developmental and evolutionary diversity of plant MADS-domain factors: insights from recent studies. Development 139, 3081–3098. 10.1242/dev.074674, PMID: 22872082

[ref60] Soltis D. E. Chanderbali A. S. Kim S. Buzgo M. Soltis P. S. (2007). The ABC model and its applicability to basal angiosperms. Ann. Bot. 100, 155–163. 10.1093/aob/mcm117, PMID: 17616563 PMC2735328

[ref002] Solovyev V. (2007). “Statistical approaches in eukaryotic gene prediction,” in Handbook of Statistical Genetics. 3rd *Edn*. eds. Balding D. J. Bishop M. Cannings C. (Wiley-Interscience), 1616. PMID:

[ref61] Theissen G. Melzer R. Rumpler F. (2016). MADS-domain transcription factors and the floral quartet model of flower development: linking plant development and evolution. Development 143, 3259–3271. 10.1242/dev.134080, PMID: 27624831

[ref62] Theissen G. Saedler H. (2001). Floral quartets. Nature 409, 469–471. 10.1038/35054172, PMID: 11206529

[ref63] Thompson B. E. Bartling L. Whipple C. Hall D. H. Sakai H. Schmidt R.. (2009). Bearded-ear encodes a MADS box transcription factor critical for maize floral development. Plant Cell 21, 2578–2590. 10.1105/tpc.109.067751, PMID: 19749152 PMC2768933

[ref64] Trevaskis B. Hemming M. N. Dennis E. S. Peacock W. J. (2007a). The molecular basis of vernalization-induced flowering in cereals. Trends Plant Sci. 12, 352–357. 10.1016/j.tplants.2007.06.010, PMID: 17629542

[ref65] Trevaskis B. Tadege M. Hemming M. N. Peacock W. J. Dennis E. S. Sheldon C. (2007b). Short vegetative phase-like MADS-box genes inhibit floral meristem identity in barley. Plant Physiol. 143, 225–235. 10.1104/pp.106.090860, PMID: 17114273 PMC1761976

[ref66] Trifinopoulos J. Nguyen L. T. von Haeseler A. Minh B. Q. (2016). W-IQ-TREE: a fast online phylogenetic tool for maximum likelihood analysis. Nucleic Acids Res. 44, W232–W235. 10.1093/nar/gkw256, PMID: 27084950 PMC4987875

[ref67] Waddington S. R. Cartwright P. M. Wall P. C. (1983). A quantitative scale of spike initial and pistil development in barley and wheat. Ann. Bot. 51, 119–130. 10.1093/oxfordjournals.aob.a086434

[ref68] Wang C. Yang X. Li G. (2021). Molecular insights into inflorescence meristem specification for yield potential in cereal crops. Int. J. Mol. Sci. 22:3508. 10.3390/ijms22147719, PMID: 33805287 PMC8037405

[ref69] Wang H. H. Zhang L. Cai Q. Hu Y. Jin Z. M. Zhao X. X.. (2015). OsMADS32 interacts with PI-like proteins and regulates rice flower development. J. Integr. Plant Biol. 57, 504–513. 10.1111/jipb.12248, PMID: 25081486

[ref70] Wei B. Zhang R. Z. Guo J. J. Liu D. M. Li A. L. Fan R. C.. (2014). Genome-wide analysis of the MADS-box gene family in *Brachypodium distachyon*. PLoS One 9:e84781. 10.1371/journal.pone.0116239, PMID: 24454749 PMC3890268

[ref71] Wu D. Liang W. Zhu W. Chen M. Ferrandiz C. Burton R. A.. (2018). Loss of LOFSEP transcription factor function converts spikelet to leaf-like structures in rice. Plant Physiol. 176, 1646–1664. 10.1104/pp.17.00704, PMID: 29217592 PMC5813523

[ref72] Wu F. Shi X. Lin X. Liu Y. Chong K. Theissen G.. (2017). The ABCs of flower development: mutational analysis of AP1/FUL-like genes in rice provides evidence for a homeotic (A)-function in grasses. Plant J. 89, 310–324. 10.1111/tpj.13386, PMID: 27689766

[ref73] Yadav S. R. Prasad K. Vijayraghavan U. (2007). Divergent regulatory OsMADS2 functions control size, shape and differentiation of the highly derived rice floret second-whorl organ. Genetics 176, 283–294. 10.1534/genetics.107.071746, PMID: 17409064 PMC1893039

[ref74] Yamaguchi T. Lee D. Y. Miyao A. Hirochika H. An G. Hirano H. Y. (2006). Functional diversification of the two C-class MADS box genes OSMADS3 and OSMADS58 in *Oryza sativa*. Plant Cell 18, 15–28. 10.1105/tpc.105.037200, PMID: 16326928 PMC1323481

[ref75] Yamaguchi T. Nagasawa N. Kawasaki S. Matsuoka M. Nagato Y. Hirano H. Y. (2004). The YABBY gene DROOPING LEAF regulates carpel specification and midrib development in *Oryza sativa*. Plant Cell 16, 500–509. 10.1105/tpc.018044, PMID: 14729915 PMC341919

[ref76] Yao S. G. Ohmori S. Kimizu M. Yoshida H. (2008). Unequal genetic redundancy of rice PISTILLATA orthologs, OsMADS2 and OsMADS4, in lodicule and stamen development. Plant Cell Physiol. 49, 853–857. 10.1093/pcp/pcn050, PMID: 18378529

[ref77] Zhu C. Yang J. Box M. S. Kellogg E. A. Eveland A. L. (2018). A dynamic co-expression map of early inflorescence development in *Setaria viridis* provides a resource for gene discovery and comparative genomics. Front. Plant Sci. 9:1309. 10.3389/fpls.2018.01309, PMID: 30258452 PMC6143762

