# Supplementary material for: Transcript Profiling of MIKCc MADS-Box Genes Reveals Conserved and Novel Roles in Barley Inflorescence Development
Source: Front Plant Sci. 2021 Sep 1;12:705286. doi: 10.3389/fpls.2021.705286 (PMC8442994; doi:10.3389/fpls.2021.705286)
Supplement: Supplementary file 1 [file Data_Sheet_1.DOCX]

**Supplementary Materials**


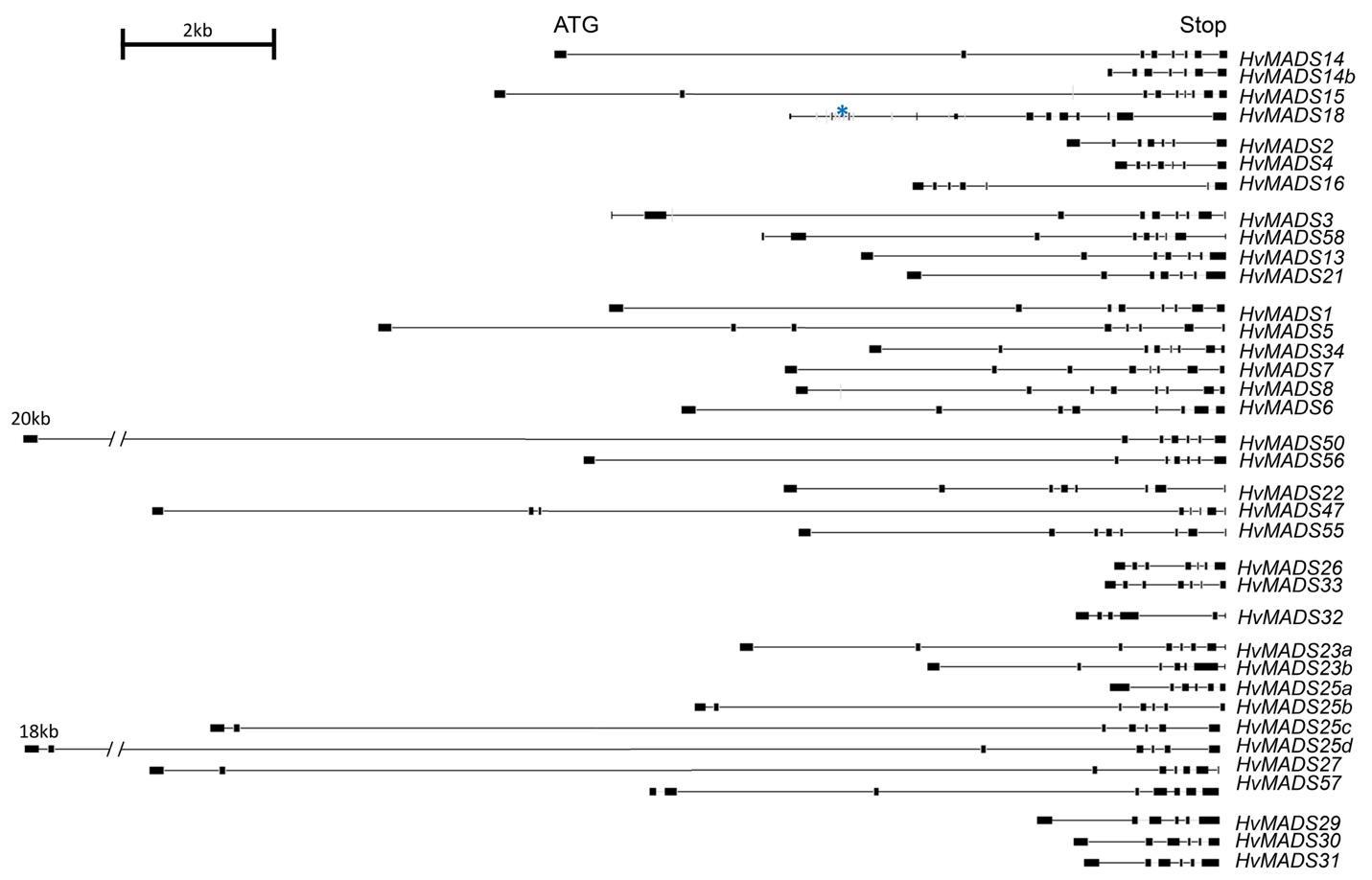
**Supplementary Figure 1**. Structure of barley MIKCc MADS-box genes, ordered by class. The scale bar indicates the length on the genome. The star at *HvMADS18* labels an unresolved region in the gene structure where the genomic sequence is incomplete. For *HvMADS50* and *HvMADS25d* the total gene length on the genome is given. Black rectangles indicate exons.


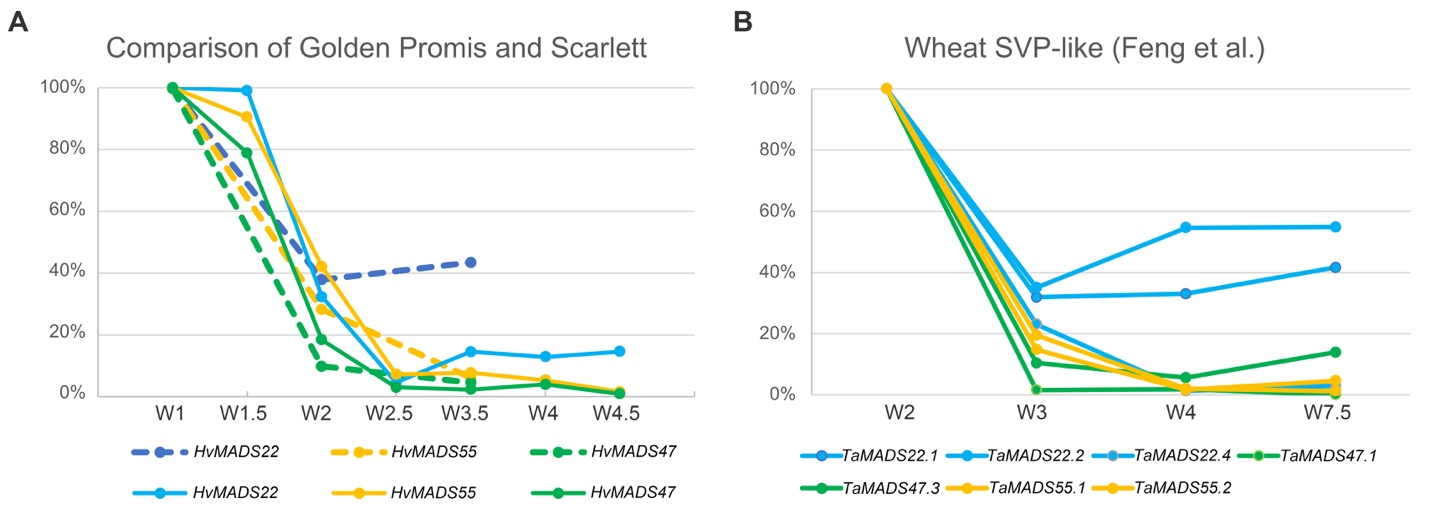


**Supplementary Figure 2**. Comparison of SVP-like gene expression between barley and wheat. (A) Comparison of SVP-like MADS-box gene expression profiles during early inflorescence development in Scarlett (Ppd-H1) (Digel et al. 2015; dashed lines) and Golden Promise (solid lines). Expression is given as a percentage of the maximum recorded expression, which is at Waddington stage 1 (W1) for all three genes from both varieties. The same pattern of a rapid decline in expression before W2, followed by a continued slower decline for *HvMADS55* and *HvMADS47*, while *HvMADS22* rebounds, is preserved. (B) Relative expression of the SVP-like genes in wheat, by Waddington stage. All expression declines after the floral transition, and the *MADS47* and *MADS55* ortholog expression remains low. Two out of three *MADS22* orthologs show a resurgence later in inflorescence development reminiscent of the expression in barley.


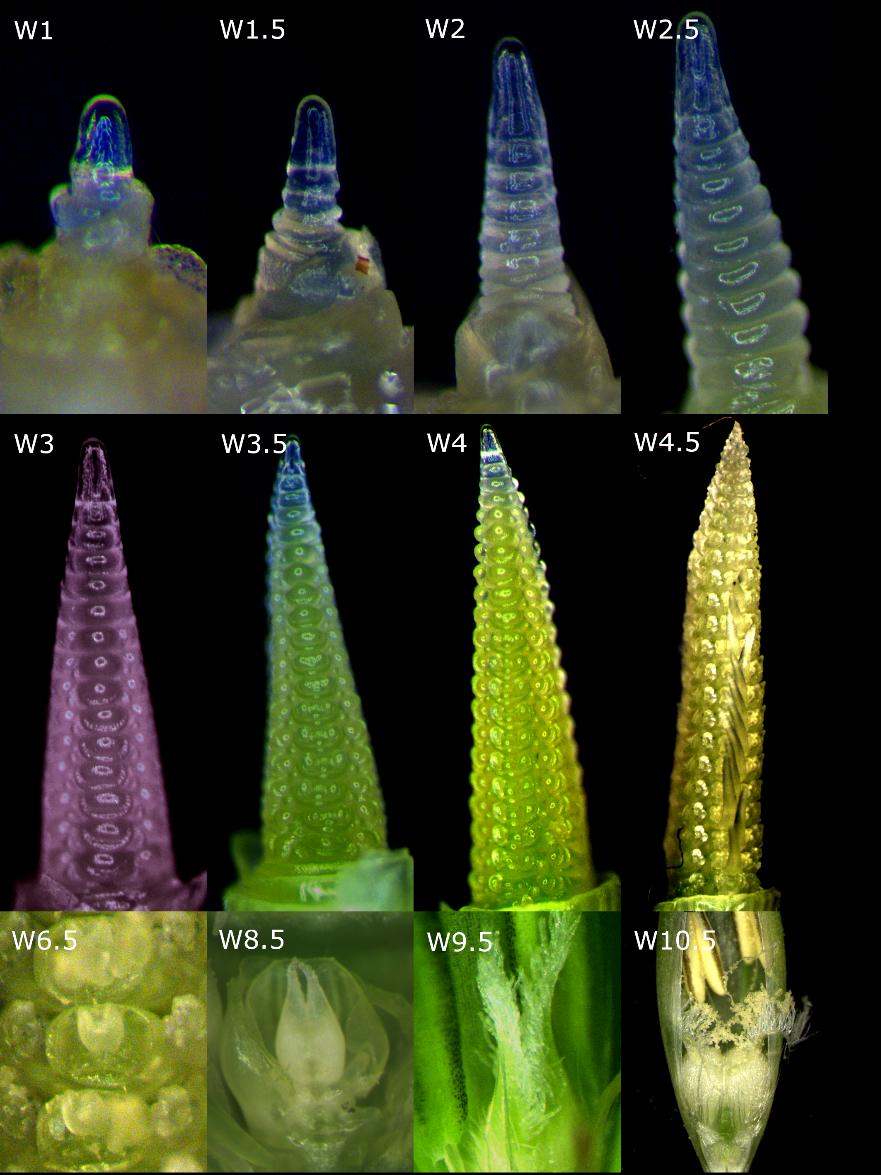


**Supplementary Figure 3**. Waddington stages as sampled. W1: vegetative meristem starting elongation, W1.5: early reproductive meristem, W2: double ridge, W2.5: triple mound (initiation of central and lateral spikelets), W3: lemma primordium, W3.5: stamen primordia, W4: pistil primordium, W4.5: carpel primordium; awn primordium; inflorescence apex diminishes (no more new spikelets are formed), W6.5: styles are prominent on the pistil (here made visible by removing the lemma and stamens), W8.5: stigmatic branches elongating and hair forms on the ovary wall, W9.5: styles and stigmatic branches are spreading, W10.5: pollen has fertilised the ovule; styles, stigmatic branches and stamens wither; palea and lemma cease growth.

**
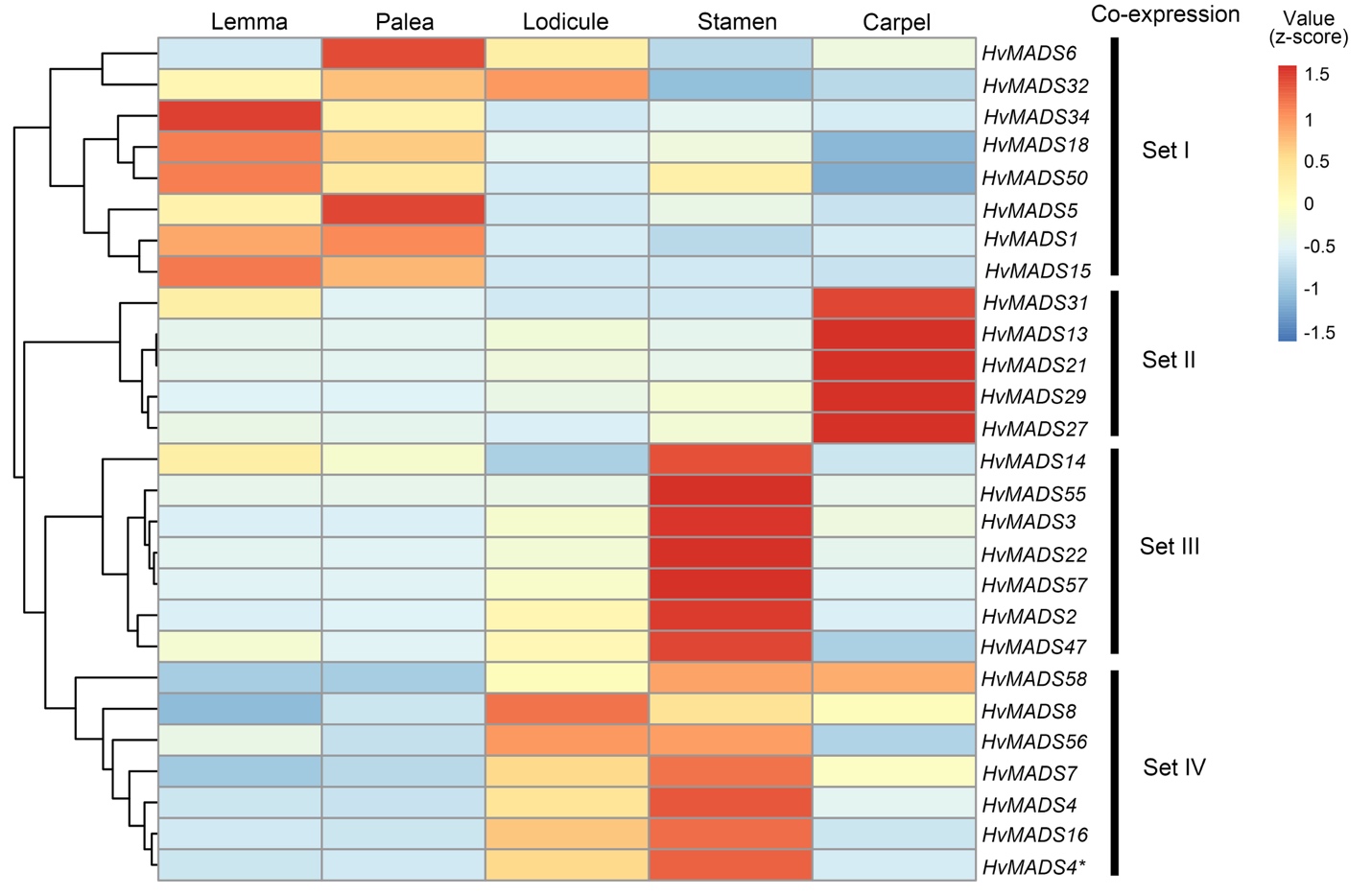
**

**Supplementary Figure 4**. Hierarchical clustering analysis of the MIKCc MADS-box gene expression in floral organs at Waddington stage 9.5. Le: lemma, pa: palea, lo: lodicules, st: stamens, ca: carpel.


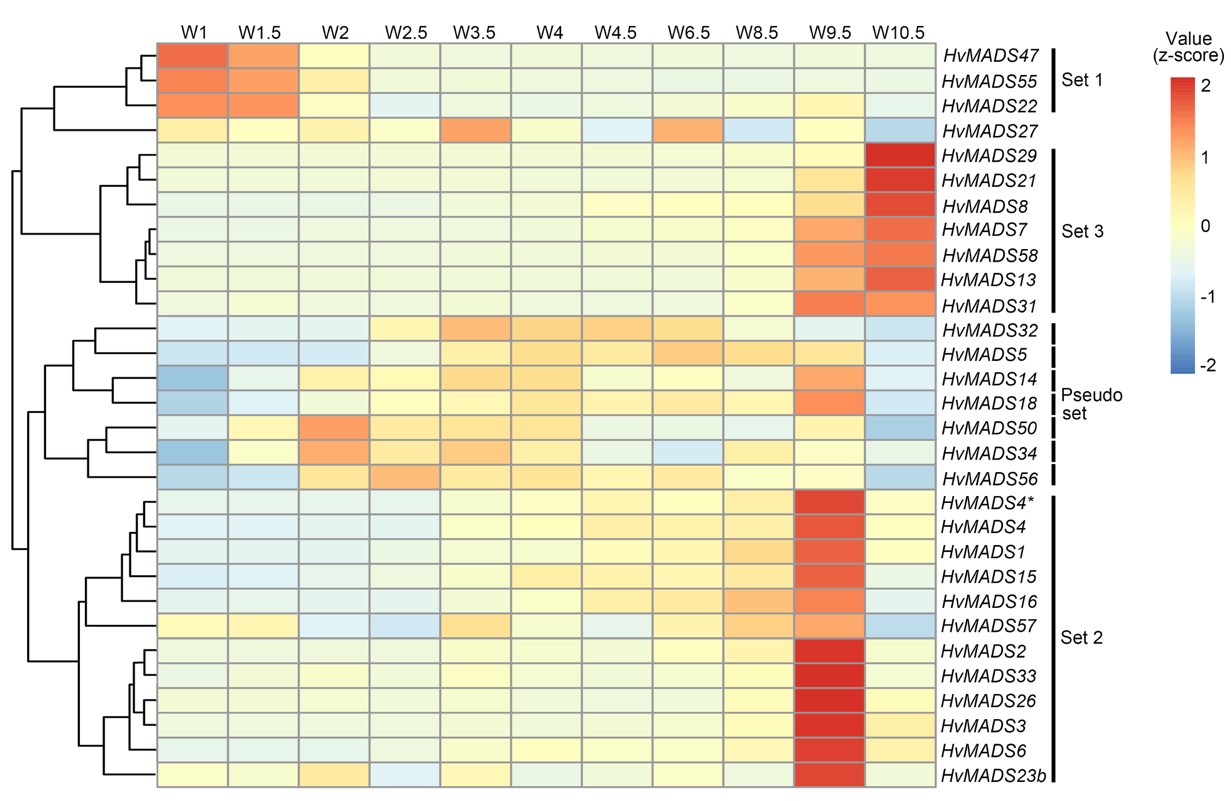


**Supplementary Figure 5**. Hierarchical clustering analysis of the MIKCc MADS-box gene transcripts during inflorescence development. The cluster was performed using the transcriptional value as the correlation groups shown in Figure 6A.

|  | Rice SNPs | Average % occurrence | SNPs in SNPbr | SNPs in Pan |  |  | No. of rice SNPs | Average % occurrence | SNPs in SNPbr | SNPs in Pan |
| --- | --- | --- | --- | --- | --- | --- | --- | --- | --- | --- |
| *MADS14* |  | - |  |  |  | *MADS56* | 4 | 25 |  | 0* |
| *MADS15* |  | - | 2 | 1 |  | *MADS22* |  | - |  |  |
| *MADS18* | 1 | 4 | 1 | 1+1 |  | *MADS55* | 1 | 3 |  |  |
| *MADS20* | 9 | 10 |  |  |  | *MADS47* | 1 | 22 | 1 | 1 |
| *MADS2* | 1 | 33 | 1 | 2 |  | *MADS26* | 1 | 7 |  | 2 |
| *MADS4* |  | - | 1 |  |  | *MADS33* | 1 | 1 |  | 3 |
| *MADS16* | 3 | 21 |  |  |  | *MADS23* |  |  |  |  |
| *MADS3* | 1 | 42 |  | 1 |  | *MADS23b* | 2 | 7 |  | 4 |
| *MADS58* | 5 | 13 |  | 0+1 |  | *MADS25a* |  |  |  | 13 |
| *MADS13* | 2 | 41 |  |  |  | *MADS25b* |  |  |  | >30 |
| *MADS21* | 2 | 4 | 1 | 0+1 |  | *MADS25c* | 1 | 27 | 2 | >30 |
| *MADS1* |  | - |  |  |  | *MADS25d* |  |  |  | 7 |
| *MADS5* | 1 | 8 |  | 2 |  | *MADS27* | 2 | 5 |  |  |
| *MADS34* |  | - |  | 2 |  | *MADS57* | 1 | 2 |  | 3# |
| *MADS7* | 2 | 43 |  |  |  | *MADS29* | 1 | 49 |  | 3 |
| *MADS8* |  | - |  |  |  | *MADS30* | 9 | 1 |  | 12 |
| *MADS6* | 1 | 17 |  | 0+1 |  | *MADS31* | 2 | 27 | 2 |  |
| *MADS50* | 3 | 16 |  | 7 |  | *MADS32* | 1 | 3 |  | 2 |

**Supplementary Table 1.** SNPs variations resulting in amino acid changes in the MIKCc genes between rice and barley. The average percentage of the rice accessions with each SNPs is given per gene. For example, two SNPs that lead to amino acid changes were found in *OsMADS7,* on average they each occurred in 43% of the cultivars, while no amino acid-altering SNPs were found in *HvMADS7.* The 3k variety set at <http://snp-seek.irri.org/_snp.zul> was used for rice, and the complete collection at [https://bridge.ipk-gatersleben.de/bridge//#snpbrowser](#snpbrowser) (“SNPbr”) and Pan-genome collection (“Pan”) for barley. +1 indicates an additional amino acid indel; *based on a partial transcript; #gene not predicted in all varieties of Pan data.

| **Gene** | **Forward primer** | **Reverse primer** |  | | |
| --- | --- | --- | --- | --- | --- |
| *MADS14* | CAGCGGCGGCAGGCGAGAG | CCAGGCTGGCCGCTGCAAC |  | | |
| *HvMADS14b* | AGCTCACTGAAAGATATCAGAT | ATCCTCTGCCCTCACGCCTAT | * | | |
| *HvMADS15* | ATATGCCTACCGCCATGGAT | ATACAGCGAACCAGCATTCC |  | | |
| *HvMADS18* | ACCAGCACAAGCAAACAACA | TGCAGGAAAAGCTCAAGACA |  | | |
| *HvMADS2* | CCAGCATGATATCGCCTTG | TCGAGCCAGTGGTGGATAA |  | | |
| *HvMADS4* | ATGCCAAGATGTTCCTGGTC | TTTGGCACCTTAGCCATCAT |  | | |
| *HvMADS16* | CGGCAAGTACCACGAGTTCT | CGTCCAGATCTTCACCCATC |  | | |
| *HvMADS3* | GCAGCAGCAGCATTACTCC | ACACATGCACGCGACAGTA |  | | |
| *HvMADS58* | ATCATGCAGCAGCCTCAGT | GGTGTGGCCAAGCCTTAAT |  | | |
| *HvMADS13* | TCAGCTGAACCTAGGCTGC | TTTGACAGGAATAGTTGAGTACTGGT | |  | |
| *HvMADS21* | CTCTTCACCTCGGCTACGA | TCTTCACAACACGCACACG |  | | |
| *HvMADS1* | TCGTCTGCAGGTTGGATATG | CAGCGTACAACGCAGCTTAG |  | | |
| *HvMADS5* | CCTGGATCACATGAACAATGA | CGAAATGCGCACATGTCTAT |  | | |
| *HvMADS34* | ATTTCGTGGCATGGATGTG | AACACAAAAGCAGCCGAGTT |  | | |
| *HvMADS7* | ACCCTCCTGAGTCCCTGAA | ACGAAAGTTGCACGCAAAA |  | | |
| *HvMADS8* | CTCAGGAGCAGATAAACAACG | GTACGCGAACGCGGTACTA |  | | |
| *HvMADS6* | CCAATAATATTCCACGGAGCA | ACGCAGGGTACTTCTCGTGT |  | | |
| *HvMADS50* | ACGCGGTTGAAGTGGAGAC | CTGCTGCTTGCAACTCCAT |  | | |
| *HvMADS56* | GGCTGTCAGGTCAATCCAA | CATCGTCGCAGCAGCTAAT |  | | |
| *HvMADS22* | TGCCATGGAAGTAACAGTAGTTG | TCGCTTCAGCTGCAAGTTT |  | | |
| *HvMADS55* | CTCGCAGGACAATGACGAC | TGCGCATTGCAGACATAAA |  | | |
| *HvMADS47* | CACCAACACGTCGTATCCA | CATCCAGTGCGCTCAAACT |  | | |
| *HvMADS26* | TGGACTGATCGACGTAGGC | AGGCCTTAAGGGCACTCCT |  | | |
| *HvMADS33* | AATTGAGCAGAGCGGGTTT | GCAATCTGCGAACTGTTCAA |  | | |
| *HvMADS23a* | TGAGCAACCACAATCACCA | CATCCCGGATTCAACACAG |  | | |
| *HvMADS23b* | GCCGGGCGATTACTAGTTC | CCGATCATCTCCCAAACCT |  | | |
| *HvMADS25a* | CATGTTATCCTCCAAGCCAGA | TCCAACTCACAGCTGACTCTGT | * | | |
| *HvMADS25b* | AGCAGCTCGAAAGTTGCTG | CATCCTCATGCTCCTGCTG | * | | |
| *HvMADS25c* | GCCAGTGGAAGCAACTCAA | TACTGCATGCTGTGGACGA | * | | |
| *HvMADS25d* | CACTGGAAGCAGCTCCAAA | GCTTGCAGTGGACGAGGTA | * | | |
| *HvMADS27* | GAGCATACTGCACCGCCTA | CGTCACATGCGAACCACTT |  | | |
| *HvMADS57* | CTCCAGCACTGGGACTTCA | AGCGCACAACATGCTACATT |  | | |
| *HvMADS29* | GAAGAGATTAACCACGAT | TCCAAGATATGCTCCTT |  | | |
| *HvMADS31* | ATGAACCCGAAGCTGTTCC | AAGCTCCGATCATCCATCC |  | | |
| *HvMADS32* | GCCTGGACCTCAAACTTGG | AGTCCAGCCCAGCCTAAAC |  | | |
| *HORVU1Hr1G063610* | TTCTCGTGTTTGTTCTGGTCA | ATGCCAAGATGTTCCTGGTC |  | | |
| *GAP* | GTGAGGCTGGTGCTGATTACG | TGGTGCAGCTAGCATTTGAGAC | Burton et al, 2008 | | |
| *Cyclophilin* | CCTGTCGTGTCGTCGGTCTAAA | ACGCAGATCCAGCAGCCTAAAG | Burton et al, 2008 | | |
| *Tubulin* | AGTGTCCTGTCCACCCACTC | AGCATGAAGTGGATCCTTGG | Burton et al, 2008 | | |
| *SP70* | CGACCAGGGCAACCGCACCAC | ACGGTGTTGATGGGGTTCATG | Burton et al, 2008 | | |
| ** did not result in usable expression data* | |  |  | |  |

**Supplementary Table 2.** Primers used for RT-qPCR.

| **Gene** | **probe** |  | **primer** |
| --- | --- | --- | --- |
| *HvMADS1* | Sense | F | TAATACGACTCACTATAGGGAGATACCGCACCTGCAACTC |
|  |  | R | GGTGTCTTGCAGCTTCTTCC |
|  | Antisense | F | AGATACCGCACCTGCAACTC |
|  |  | R | TAATACGACTCACTATAGGGGGTGTCTTGCAGCTTCTTCC |
| *HvMADS6* | Sense | F | TAATACGACTCACTATAGGGGGCACAACAAAAACATTGGA |
|  |  | R | GTGCTTGAGTTGCCTGTTGA |
|  | Antisense | F | GGCACAACAAAAACATTGGA |
|  |  | R | TAATACGACTCACTATAGGGGTGCTTGAGTTGCCTGTTGA |

**Supplementary Table 3.** Primers for the generation of *in situ* hybridisation RNA probes.


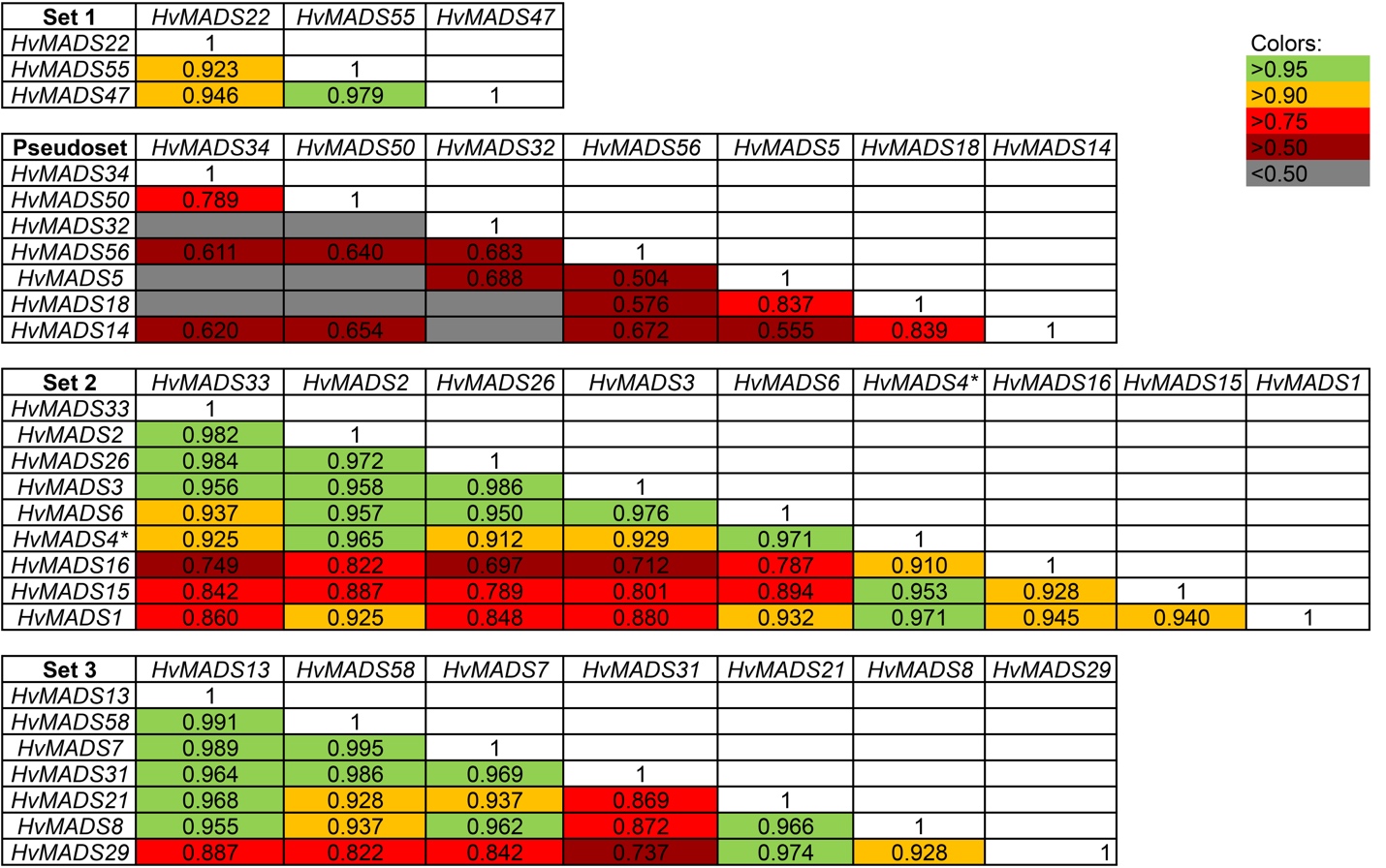


**Supplementary Table 4**. Individual correlation score table for set 1, set 2, set 3 and the pseudoset. Green backgrounds indicate correlation over 0.95.
